# The SWIB domain-containing DNA topoisomerase I of *Chlamydia trachomatis* mediates DNA relaxation

**DOI:** 10.1128/jb.00190-25

**Published:** 2025-08-12

**Authors:** Li Shen, Abigail R. Swoboda, Caitlynn Diggs, Shomita Ferdous, Andrew Terrebonne, Amanda Santos, Noel Wolf, Luis Lorenzo Carvajal, Guangming Zhong, Scot P. Ouellette, Yuk-Ching Tse-Dinh

**Affiliations:** 1 Department of Microbiology, Immunology, and Parasitology, Louisiana State University Health Sciences Center, New Orleans, Louisiana, USA; 2 Department of Pathology, Microbiology, and Immunology, University of Nebraska Medical Center 12284, Omaha, Nebraska, USA; 3 Department of Chemistry and Biochemistry, Florida International University 5450, Miami, Florida, USA; 4 Department of Microbiology, Immunology and Molecular Genetics, University of Texas Health San Antonio, San Antonio, Texas, USA; University of Notre Dame 6111, Notre Dame, Indiana, USA

**Keywords:** *Chlamydia trachomatis*, DNA topoisomerase I (TopA), DNA relaxation, SWIB domain, transcriptional regulation, chlamydial developmental cycle, CRISPRi

## Abstract

*Chlamydia trachomatis* has a DNA topoisomerase I with a unique C-terminal domain (CTD) homologous to eukaryotic SWIB domains. This study focused on determining the function of the SWIB domain-containing TopA from *C. trachomatis* (CtTopA). We demonstrated that, despite the lack of sequence similarity at the CTDs between CtTopA and TopA from *Escherichia coli* (EcTopA), full-length CtTopA removed negative DNA supercoils *in vitro* and complemented the growth defect of a *topA* mutant of *E. coli*. CtTopA is less processive in DNA relaxation than EcTopA in dose-response and time course studies. An antibody generated against the SWIB domain of CtTopA specifically recognized CtTopA but not EcTopA or *Mycobacterium tuberculosis* TopA, consistent with the sequence differences in their CTDs. The endogenous CtTopA protein is expressed at a relatively high level during the middle and late developmental stages of *C. trachomatis*. Overexpressing a *topA* mutant allele lacking the SWIB domain in *C. trachomatis* resulted in slow growth when host protein synthesis was inhibited. These data suggest that productive infection of *C. trachomatis* requires functional SWIB domain-containing CtTopA and *de novo* host protein synthesis. Because SWIB domain-containing CtTopAs are not found in prokaryotes beyond *Chlamydia* spp., our work suggests an important function of the SWIB domain on CtTopA activity during *C. trachomatis* infection.

**IMPORTANCE:**

*Chlamydia trachomatis* is a medically important bacterial pathogen that is responsible for the most prevalent bacterial sexually transmitted infection. Bioinformatics, genetics, and biochemical analyses have established that the presence of a SWIB domain in CtTopA is relevant to chlamydial physiology. Our findings also underline the mechanistic diversity among the family of TopAs that are likely driven by pathogen-specific adaptations.

## INTRODUCTION

DNA topoisomerases (Topos) are essential enzymes regulating DNA topology in all cells (1–3). Depending on their actions on DNA, Topos can be broadly divided into type I and type II. Type I Topos (e.g., TopA or TopoI) transiently cleave and reseal a single strand of the DNA helix in the absence of ATP. Type II Topos (e.g., DNA gyrase and TopoIV) cut and religate both DNA strands in the presence of ATP. In *Escherichia coli*, DNA supercoiling is chiefly balanced by DNA-relaxing TopA and the negative supercoiling-introducing DNA gyrase. The main function of TopoIV is to disentangle replicated DNA, enabling the segregation of duplicated chromosomes. Bacterial type II Topos are validated targets of fluoroquinolone antibiotics. With the alarming rise of antibiotic resistance, great efforts have been made to develop novel poisons or catalytic inhibitors of Topos to battle against difficult-to-treat infections (4, 5), including drug-resistant *Mycobacterium tuberculosis* and *Neisseria gonorrhoeae*, which often co-infect with *Chlamydia trachomatis* (6).

*C. trachomatis* is an obligate intracellular bacterium and the leading cause of bacterial sexually transmitted infections (7, 8). In 2020, an estimated 128.5 million new *C. trachomatis* cases occurred worldwide among individuals aged 15–49 years (9, 10). Over 50% of men and >75% of women with *C. trachomatis* infection are asymptomatic. The lack of durable immunity in most individuals can result in recurrent or chronic *C. trachomatis* infection. In women, this can lead to pelvic inflammatory disease and eventually to tubal factor infertility and ectopic pregnancy. Although *C. trachomatis* infection can be cured by antibiotics, a compelling whole genome sequence study indicated that *C. trachomatis* can establish chronic infections even with repeated antibiotic treatments via unknown mechanisms (11). There is an urgent need for improved strategies to solve the problems associated with *C. trachomatis* infection.

*Chlamydia* has evolved to have a small (~1 Mbp) and AT-rich chromosome (12) as a result of its long-term adaptation to the intracellular niche. After infecting mucosal epithelial cells, *C. trachomatis* lives in a host-derived parasitophorous vacuole (termed an inclusion) and varies between morphologically and functionally divergent forms that differentiate between them at early and late stages of the developmental cycle. The elementary body (EB) is a small, infectious, metabolically limited form with highly condensed chromatin, while the reticulate body (RB) is a replicative and more metabolically active form with dispersed chromatin. The observations that the *C. trachomatis* developmental cycle correlates to temporal gene expression (13, 14) and differential DNA supercoiling density (15–17) have led to the hypothesis that DNA supercoiling is a global regulator of chlamydial development. In support of this idea, *in vitro* studies have shown that selected early- and mid-developmental cycle promoters of *C. trachomatis* are supercoiling sensitive (17–19). We recently utilized CRISPRi for conditional repression of *C. trachomatis topA* to bypass lethality issues associated with the disruption of essential genes (20). Our results indicated that *C. trachomatis* with *topA* knocked down exhibited impaired growth and a greater sensitivity to fluoroquinolone antibiotics, highlighting the importance of CtTopA in the chlamydial developmental cycle.

It remains unknown how CtTopA acts to affect *C. trachomatis* physiology. Since 1998, when the first *Chlamydia* genome was published, it has been predicted that the canonical conserved catalytic domains of CtTopA are fused to a C-terminus eukaryotic SWIB domain (12). In eukaryotes, the SWIB domain is primarily found in the SWI/SNF complex B (SWIB) family, notably in BAF60b proteins involved in chromatin remodeling (21). This motif is homologous to the p53-binding domain of the MDM2 oncoprotein, suggesting a shared functional mechanism and structure engaging protein-protein interactions (22, 23). However, the SWIB domain is rarely found in prokaryotes except for *Chlamydia* spp., and its significance in these species is unknown.

The current study was focused on functional aspects of the SWIB domain-containing TopA. First, we analyzed the domain composition of CtTopA *in silico*. Second, we determined the DNA relaxation capacity of CtTopA compared to that of EcTopA *in vitro* and the ability of CtTopA, or a mutant isoform lacking the SWIB domain, to complement *E. coli* strains defective in *topA*. Third, we measured CtTopA’s expression levels in the context of infection. Finally, we tested whether overexpression of a *topA* truncation mutant with deletion of the SWIB domain affected chlamydial growth. Our findings indicate a role of the SWIB domain in CtTopA’s activity that is required for *C. trachomatis* to survive and multiply intracellularly.

## RESULTS

### The C-terminal SWIB domain of TopA is unique to *Chlamydia* spp.**—**bioinformatics evidence

**Fig 1:**
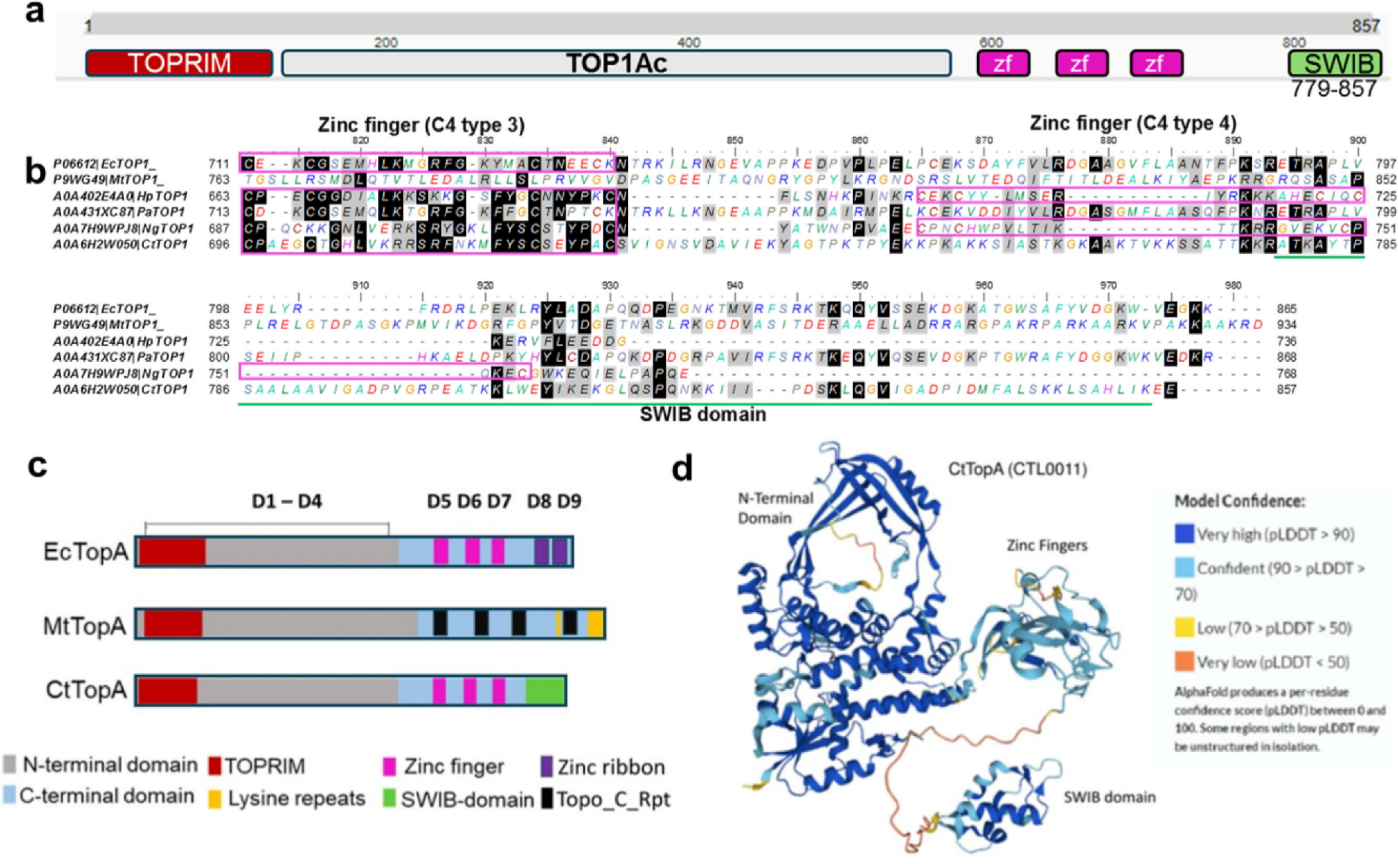
C-terminal SWIB domain is unique in CtTopA. (**a**) Domain composition of CtTopA (CTL0011) predicted by InterPro. Zf: 4C zinc fingers. The 8.5 kDa SWIB-like domain consists of 79 amino acids (green). (**b**) Alignment of amino acid residues of the CTDs of TopAs from *E. coli, M. tuberculosis*, *H. pylori*, *P. aeruginosa*, *N. gonorrhoeae,* and *C. trachomatis*. Accession numbers are shown on the left. The conserved 4C zinc fingers are boxed. The position of the SWIB domain in CtTopA is underlined (green). ClustalW was used for alignment with Matrix BLOSUM62. See Fig. S1 for entire sequence alignments of these bacterial TopAs. (**c**) Schematic diagram showing domains of the EcTopA (**D1–D9**) compared to domains found in MtTopA and CtTopA. The gray or light blue bar represents the N- and C-terminal domains. The TOPRIM (red), zinc finger (cyan), Topo_C-Rpt (black), lysine repeats (yellow), and SWIB domain (green) are as indicated. (**d**) Structural model of CtTopA by AlphaFold. The NTD, CTD zinc fingers, and the SWIB domain are as indicated. Model confidences are shown on the right.

There are two important functional domains in bacterial TopAs: the conserved N-terminal domains (NTDs), which have DNA cleavage and religation activities, and diverse C-terminal domains (CTDs), which are crucial for the ability to relax DNA and exert other catalytic activities through protein-protein interactions (1–3). To investigate CtTopA’s domain composition, we used InterPro (24), and the sequences of full-length CtTopA (consisting of 857 amino acid residues) were specifically aligned with those of TopA orthologs in *E. coli, Mycobacterium tuberculosis*, *Helicobacter pylori*, *Pseudomonas aeruginosa*, and *N. gonorrhoeae*. As shown in Fig. S1 and Fig. 1a through c, the NTDs of TopAs (corresponding to EcTopA domains 1–4 [D1–D4]) are analogous in containing the topoisomerase-primase domain (TOPRIM) and several DNA-binding sites. The TopAs’ CTDs (corresponding to EcTopA D5–D9) were highly divergent (25). Whereas the EcTopA has three four-cysteine-(4C)-zinc-finger (D5–D7) motifs and two zinc ribbon-like motifs (D8–D9) at its CTD, different numbers of the 4C-zinc-finger motifs were present in TopAs from *C. trachomatis* (three), *P. aeruginosa* (three), *H. pylori* (four), and *N. gonorrhoeae* (four). None was found in TopA from *M. tuberculosis*, which instead had four Topo_C_Repeat (Rpt) motifs and two lysine repeats. Specifically, CtTopA stands out for possessing an 8.5 kDa (79 amino acids) SWIB-like domain at its CTD (in the place of EcTopA zinc ribbon-like domains D8–D9). AlphaFold prediction determined that these amino acid sequences folded into a SWIB-like three-dimensional shape (Fig. 1d) distinct from other known structures of TopA proteins (2, 26), suggesting a potentially novel protein fold or functional variation in the TopA family. Of note, orthologs of SWIB domains were found in members of the Chlamydiaceae family we inspected (Table S1) using Basic Local Alignment Search Tool, BLASTp. Thus, CtTopA is distinguishable from other bacterial TopAs by its SWIB domain at the CTD, suggesting potentially unique functions for this TopA ortholog.

### Recombinant CtTopA catalyzes DNA relaxation *in vitro*

**Fig 2:**
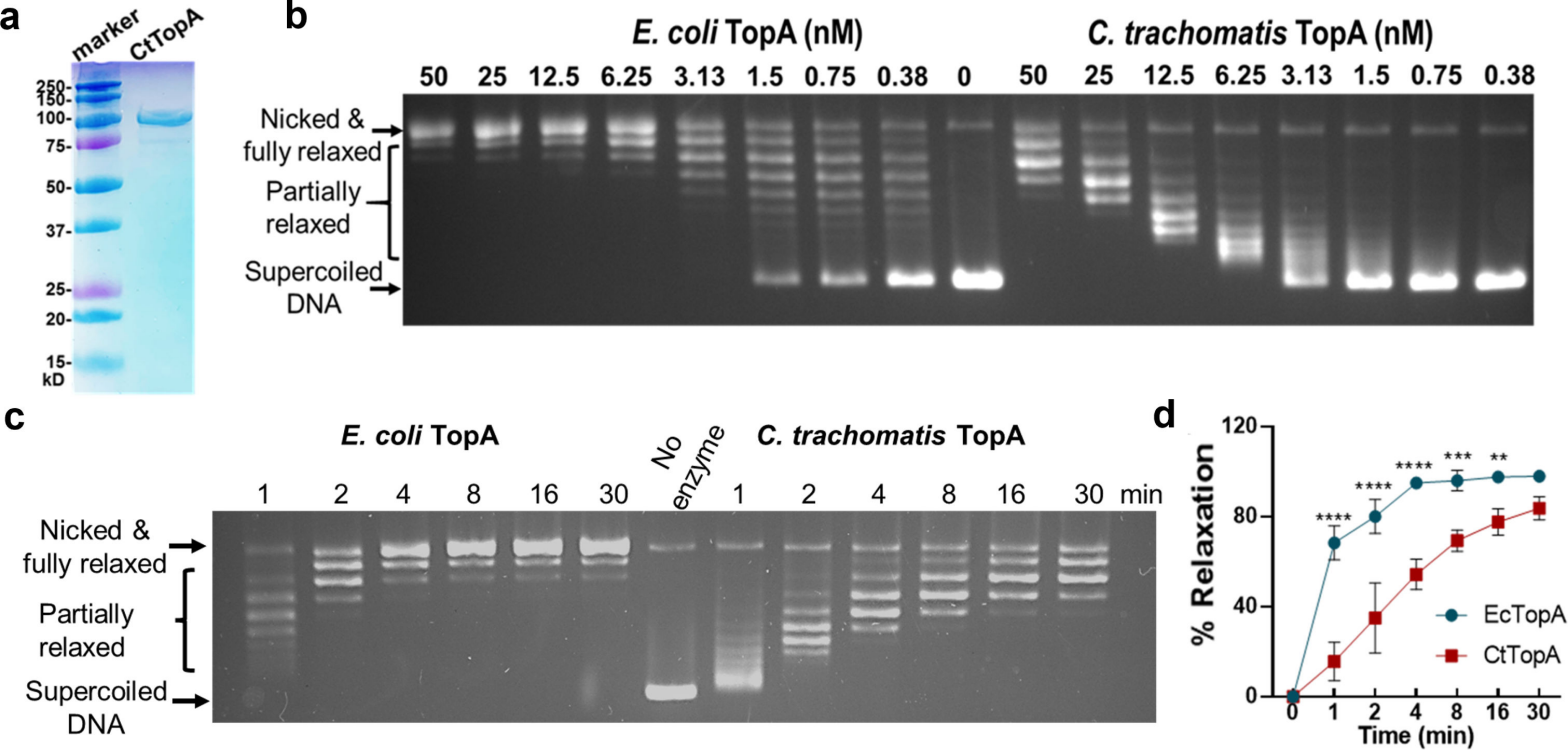
Comparison of the *in vitro* DNA relaxation activity of recombinant CtTopA to EcTopA. (**a**) SDS-PAGE/Coomassie staining gel showing recombinant CtTopA protein purified from *E. coli*. (**b**) Concentration-dependent DNA relaxation. Serial dilutions of EcTopA and CtTopA as indicated were incubated with 0.3 µg (5.2 nM) negatively supercoiled DNA for 30 min, followed by agarose gel electrophoresis. (**c**) Time course of DNA relaxation. EcTopA or CtTopA (25 nM) was incubated with 0.3 µg of negatively supercoiled DNA for different times (1–30 min). (**d**) Quantification of DNA relaxation based on time-course studies. The percentage of relaxation was determined by dividing the distance between the negatively supercoiled band (SC) and the weighted center of the partially relaxed band (PR) by the distance between the SC and the fully relaxed band (FR). Formula: percent relaxation = (SC − PR)/(SC − FR) × 100 (30). The values are reported as mean ± standard deviation of results obtained from three independent experiments (also see Fig. S4). Statistical comparison between EcTopA and CtTopA at a given time was analyzed by *t* test. **P < 0.01, ***P < 0.001, and ****P < 0.0001.

To determine the activity of CtTopA in DNA relaxation *in vitro*, we compared it with the well-studied EcTopA (1–3). To avoid potential negative impacts of the 6xHis-tag on the CTD, we designed an N-terminal 6xHis-tagged CtTopA construct under the control of an isopropyl β-D-1-thiogalactopyranoside (IPTG)-inducible T7 promoter. An N-terminal 6xHis tag has been shown not to affect the activity of EcTopA (27). After transformation of this construct into *E. coli* BL21(DE3), the CtTopA protein expression was induced by adding IPTG, purified to homogeneity (Fig. 2a), and used for DNA relaxation assays. Recombinant EcTopA was obtained as described (28, 29). With serial dilutions of CtTopA or EcTopA (at concentrations ranging from 0 to 50 nM) and constant amounts of negatively supercoiled plasmid DNA, we observed different patterns of DNA relaxation. More CtTopA protein was required for the DNA substrate to reach a fully relaxed state (Fig. 2b) at the end of the 30-min incubation period. Additionally, a time-course study was performed by incubating 25 nM of CtTopA or EcTopA with constant amounts of plasmid DNA for 0–30 min (Fig. 2c). The reaction products from CtTopA relaxation have fewer bands in the gel, corresponding to the entire population of plasmids having a similar number of superhelical turns removed during the relaxation reaction (Fig. 2c and d). This is likely because CtTopA dissociates more readily from the DNA substrate after removing each superhelical turn. Longer incubation times were required for the DNA substrate to reach a fully relaxed state as reflected by measurement of the percentage of DNA relaxation (Fig. 2d; Fig. S2). In contrast, the EcTopA relaxation activity is more processive, with the enzyme staying bound to the plasmid substrate to remove nearly all the superhelical turns instead of dissociating from the DNA substrate after removing only a few superhelical turns (Fig. 2b). These results imply that CtTopA is less efficient than EcTopA in the relaxation of negatively supercoiled DNA.

### CtTopA functionally complements *E. coli* strains with *topA* mutations

**Fig 3:**
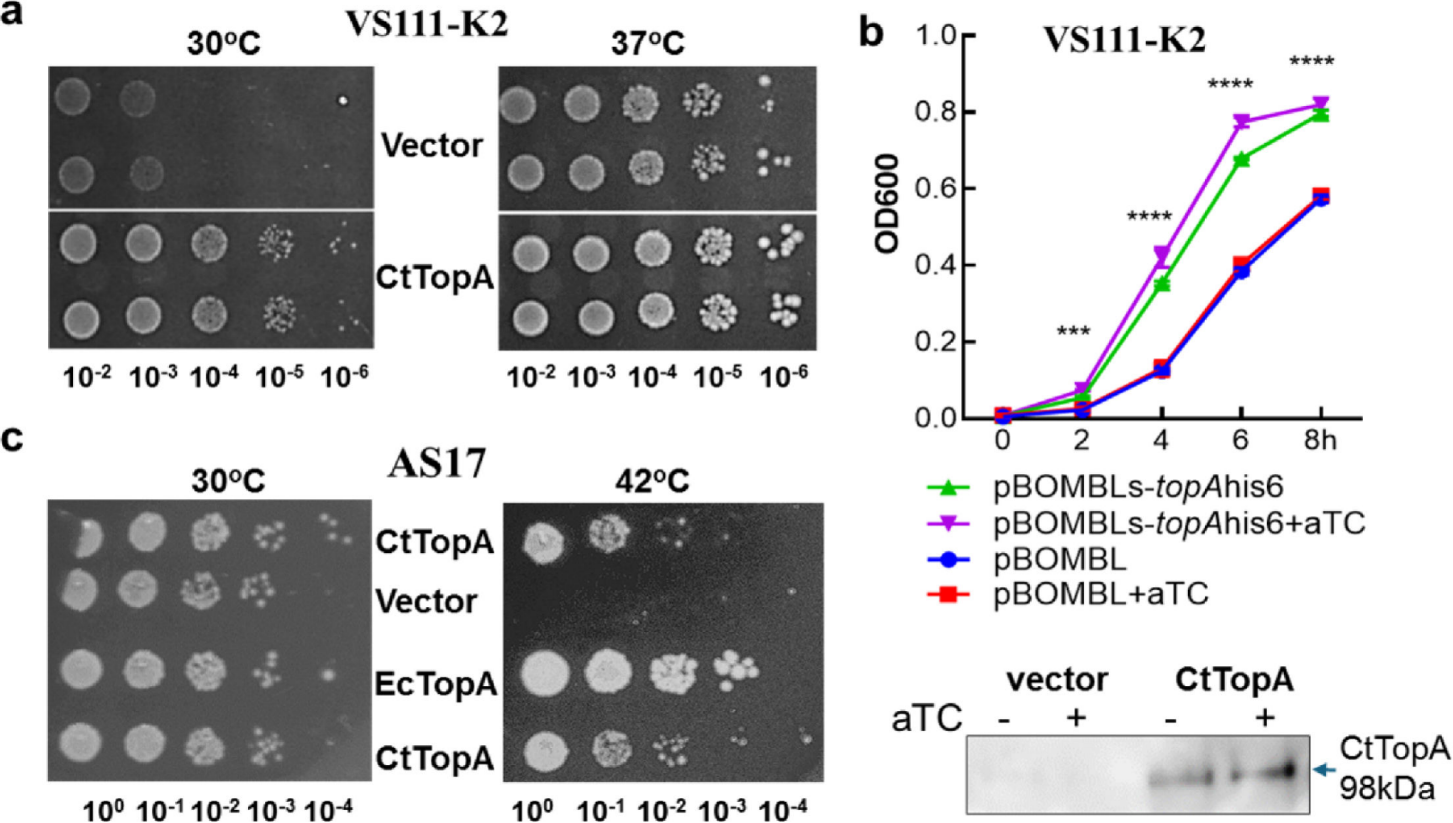
Complementation assay in *E. coli topA* mutant strains. (**a and b**) Results with strain VS111-K2 transformed with pBOMLs-*topA*His6 expressing CtTopA or vector pBOMBLs expressing mcherry. Tenfold serial dilutions of the bacterial cultures were spotted on LB agar plates containing chloramphenicol and spectinomycin. Images were taken at 18 h after incubation at 30°C or 37°C (**a**). Growth curve of *E. coli* strains as indicated during 8-h incubation at 37°C in the presence or absence of aTC at 200 µg/mL (b). *Y*-axis: OD_600_, *x*-axis: hours of incubation. Data are presented as mean ± SEM. (n=3) Statistical comparisons of OD_600_ between induced and uninduced samples of the same strain were performed by two-way ANOVA. ***P < 0.001 and ****P < 0.0001. Lower panel: immunoblotting showing expression of His6-tagged CtTopA in *E. coli* with anti-His antibody. Note: leaky expression of CtTopA in the absence of aTC. (**c**) Results with AS17 transformed with pLIC-EcTOP expressing EcTopA or pET-CtTopA expressing CtTopA as indicated. Tenfold serial dilutions of the cultures of the transformants were spotted on LB agar plates with kanamycin and incubated at 30°C or 42°C. Images were taken after 18 h for 42°C incubation and 36 h for 30°C incubation. For all strains, two different isolates of *E. coli* transformants were used as biological replicates.

Next, we examined CtTopA function in *E. coli topA* mutant strains (Table S2). *E. coli* VS111-K2 (31) is cold sensitive and has a growth defect at 30°C due to the *ΔtopA* mutation resulting in excessive negative DNA supercoiling. If CtTopA functions in *E. coli*, then bacterial growth should be improved at 30°C. We transformed a pBOMB-based shuttle plasmid (20) expressing *tet* promoter (P*_tet_*)-controlled *C. trachomatis topA* or mCherry as a vector control into *E. coli* VS111-K2. After incubation on LB agar plates at 30°C for 18 h, the CtTopA-expressing strain exhibited better growth than the vector control strain regardless of the addition of inducer anhydrotetracycline (aTC) (Fig. 3a), suggesting possible leaky expression of CtTopA in uninducing conditions. This was confirmed by immunoblotting for CtTopA in *E. coli* (Fig. 3b). Both strains grew well at 37°C, as expected. The capacity of CtTopA to complement was also studied by a growth curve assay at 37°C (Fig. 3b). We observed that the growth of the CtTopA-expressing strain was greatly improved compared to the vector control.

To further evaluate the complementing efficiency, we used *E. coli* strain AS17 (32–34). The *topA* gene in AS17 has a G65N mutation and an amber codon instead of the W79 residue found in wild-type EcTopA. Studies have shown that AS17 is not viable for growth at 42°C because of a lack of relaxation activity from the chromosomally encoded EcTopA at the non-permissive temperature (32, 33). However, background noninduced expression of bacterial Topo I under the control of the T7 promoter in an expression plasmid can complement the growth of *E. coli* AS17 at 42°C (33). We transformed the plasmid pET28-CtTopA, expressing *C. trachomatis topA,* or pLIC-EcTOP, expressing *E. coli topA* controlled by a T7 promoter (28), into AS17. Strains with the corresponding empty vector were used as negative controls. We observed that the CtTopA-expressing clone supported the growth of *E. coli* AS17 at 42°C (Fig. 3c). Compared to the EcTopA-expressing positive control, the CtTopA clone grew at about 10-fold lower efficiency, consistent with the less robust relaxation activity for CtTopA in the *in vitro* enzyme activity assay (Fig. 2b through d). These results indicate that expression of basal levels of CtTopA is necessary and sufficient to correct the growth defect of *E. coli topA* mutants.

### Antibodies specifically recognize the CtTopA protein

**Fig 4:**
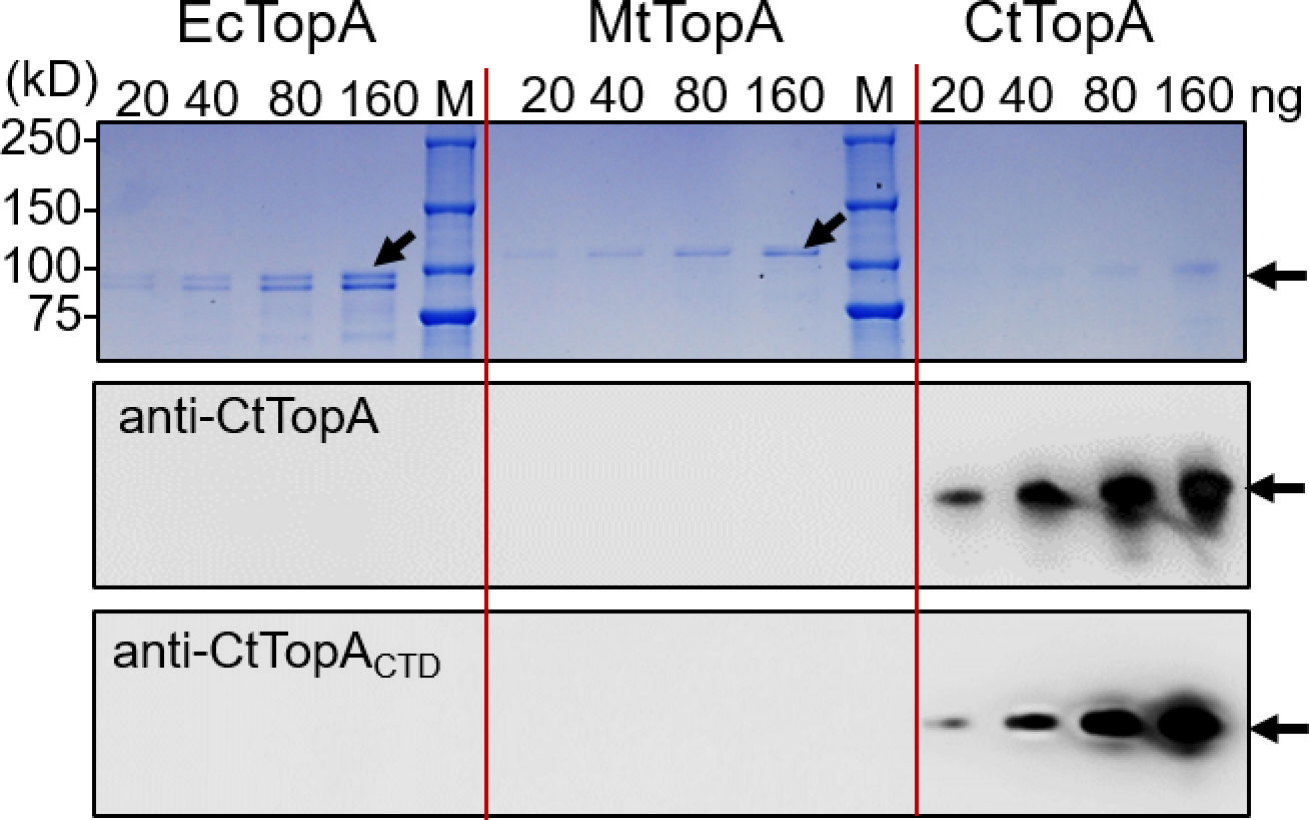
Reaction of anti-CtTopA or anti-CtTopA_CTD_ with the purified recombinant CtTopA. Serial dilutions of EcTopA, MtTopA, and CtTopA proteins on SDS-PAGE/Coomassie-stained gel (upper panel) and immunoblots showing their reactions to anti-CtTopA (middle panel) or anti-CtTopA_CTD_ (lower panel). Arrows show protein bands of interest.

To better study the domains of CtTopA and develop novel resources, we produced polyclonal antibodies using two different strategies. First, recombinant full-length CtTopA was used as the source of antigen to immunize mice, resulting in anti-CtTopA. Second, we designed and used synthesized peptides containing CtTopA amino acids 737–756 and 843–857 to co-immunize rabbits, resulting in anti-CtTopA_CTD_. Western blot analysis showed that both anti-CtTopA and anti-CtTopA_CTD_ specifically recognized an antigen corresponding to ~98 kDa recombinant CtTopA but not EcTopA or MtTopA (Fig. 4). Thus, at least one epitope recognized by both anti-CtTopA and anti-CtTopA_CTD_ is situated on the CTD of CtTopA and is not present in EcTopA and MtTopA. These data are in line with the sequence alignment (Fig. 1; Fig. S1) showing differences in the CTDs among CtTopA, EcTopA, and MtTopA.

### TopA mutant with deletion of the SWIB domain retains complementation ability** in** *E. coli* AS17

**Fig 5:**
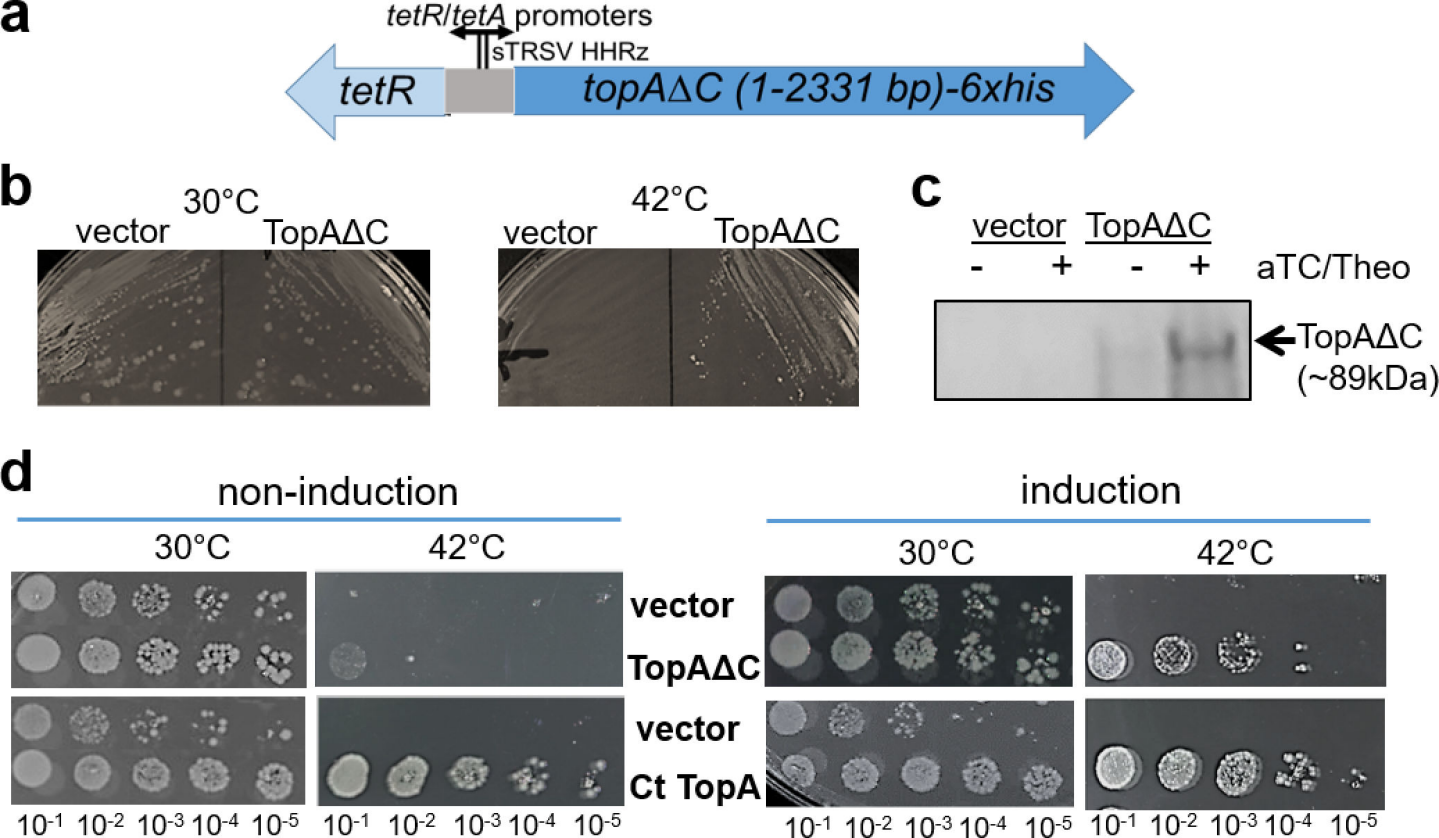
Overexpression of mutant TopAΔC complements the growth defect of *E. coli* AS17 at 42°C. (**a**) Schematic map of the construct encoding P*_tet_*/riboswitch-controlled mutant *topAΔC* (containing nucleotides 1–2,331 of chlamydial *topA* and 6xHis). (**b**) Complementation of *E. coli* AS17 with *topA^ts^* chromosomal mutation for growth at 42°C by background levels of mutant TopAΔC. Images were taken 18 h after incubation at 30°C or 37°C. (**c**) Immunoblot displays inducible expression of TopAΔC in AS17 grown in LB broth containing spectinomycin (50 µg/mL) at 30°C after the addition of aTC (200 µg/mL) and Theo (15 µg/mL) for 4 h. Anti-CtTopA was used. (**d**) Comparison of the growth of strains with CtTopA, TopAΔC, or vector control on LB agar at 30°C and 42°C under non-induction or induction conditions. Tenfold serial dilutions of the cultures of the transformants as indicated were spotted on LB agar plates with spectinomycin (50 µg/mL) and incubated at 30°C for 36 h or 42°C for 18 h. The representative plate images from three experiments are shown.

To determine whether the SWIB domain affected CtTopA activity, we created a plasmid encoding a SWIB-deleting mutant allele, *topAΔC*, with *6xHis* controlled by P*_tet_* and the theophylline (Theo)-dependent riboswitch (Fig. 5a). This allowed us to express a truncated TopA protein (amino acid residues 1–778)-tagged with 6xHis under tight control. We evaluated the effect of expressing CtTopA lacking its SWIB domain in *E. coli* AS17, which is not viable at the non-permissive temperature of 42°C but is viable when wild-type CtTopA is overexpressed (Fig. 3c). The strain carrying a mCherry-expressing vector was used as a control. The transformants with *topAΔC* plasmid survived at both 30°C and 42°C, while the vector control only grew at 30°C (Fig. 5b), indicating that basal levels of TopAΔC were able to complement the growth defect of *E. coli* AS17 at the non-permissive temperature. Immunoblots indicated the inducible expression of TopAΔC in this strain by adding aTC and Theo (Fig. 5c). A more quantitative analysis was performed to compare the growth impact of TopAΔC with that of CtTopA using spot assays. Under non-induction conditions, no significant growth differences were observed between the strains with TopAΔC and CtTopA at 30°C (Fig. 5d). Conversely, at 42°C, the TopAΔC mutant strain showed growth at a high concentration, while the CtTopA supported growth better, consistent with its leaky expression in the absence of inducer (Fig. 3). Under inducing conditions, all three strains could grow at 30°C, as expected. The strains expressing CtTopA or TopAΔC grew at 42°C; however, CtTopA had ~10-fold better complementation for growth than TopAΔC. Dot blots indicated that TopAΔC was present at approximately the same level as CtTopA (Fig. S5). The phenotypic effects of TopAΔC in *E. coli* AS17 compared with CtTopA suggest a potential role of the SWIB domain in CtTopA’s relaxation activity.

### *C. trachomatis* naturally produces SWIB domain-containing CtTopA during infection

**Fig 6:**
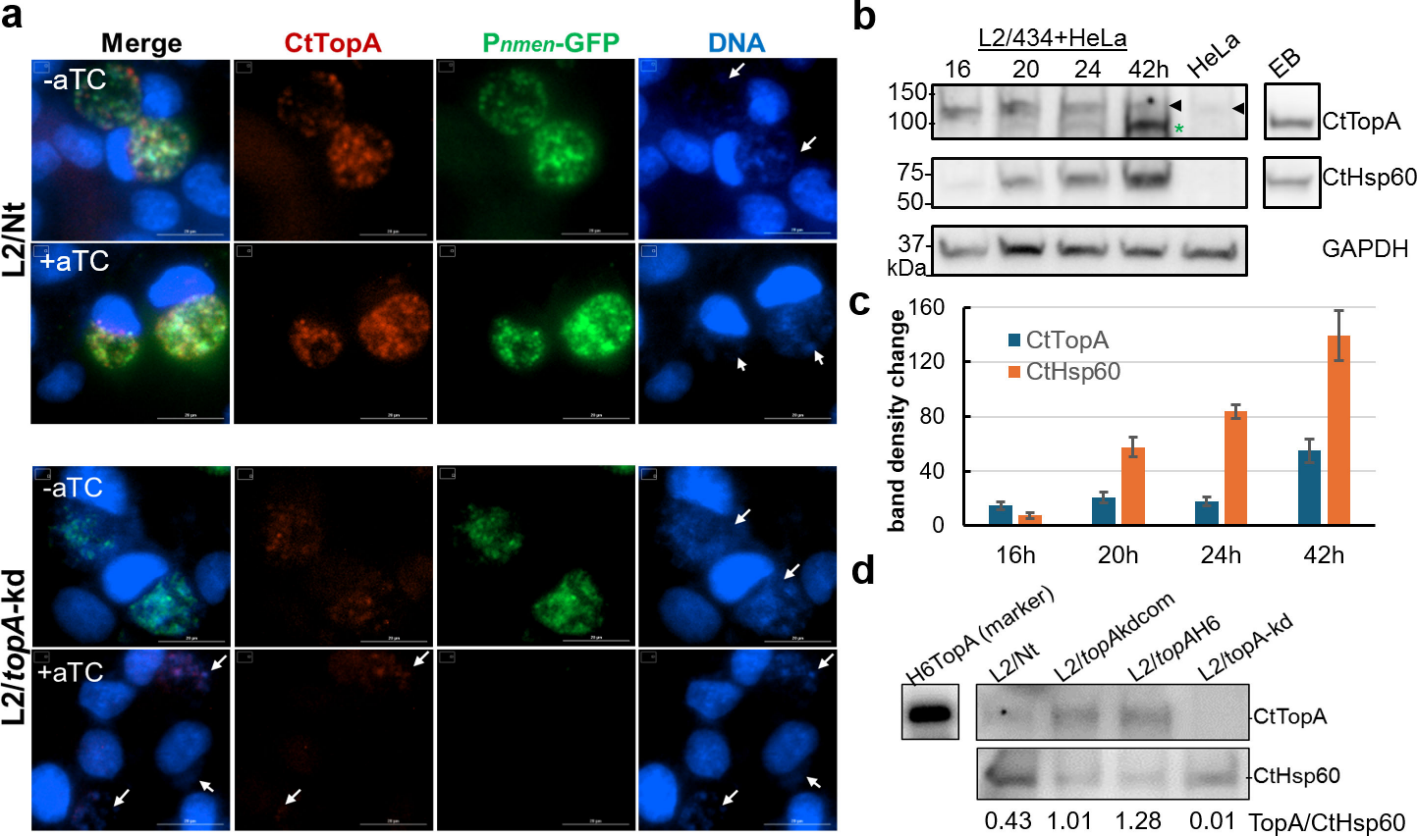
*C. trachomatis* naturally produces SWIB domain-containing CtTopA. (**a**) Immunofluorescence micrographs of HeLa cells infected with L2/Nt or L2/*topA*-kd at 45 hpi. GFP-expressing chlamydial organisms (green) were stained for CtTopA (red; anti-CtTopA antibody). Cellular and bacterial DNA was counterstained with 4′,6-diamidino-2-phenylindole dihydrochloride (blue). Arrows indicate the location of chlamydial inclusions. Left panels show merged images. Image adjustments of *C. trachomatis* and DNA were applied equally for both bacterial strains and cells. Scale bars = 20 µm. (**b and c**) Immunoblotting of endogenous chlamydial Hsp60 and CtTopA levels in lysates of infected HeLa cells sampled at 16, 20, 24, and 42 hpi. Host GAPDH was used as a loading control. *: band corresponding to ~97 kDa CtTopA. Arrow: a larger band. Densitometry of the protein band of interest was assessed using ImageJ and presented in (**C**. A representative blot is shown with the quantification of the amount relative to that of CtHsp60. Results shown are the mean ± SD (*n* = 2). Note: the level of CtTopA was significantly increased from 24 to 42 h (*t* test, *P* < 0.05). The full-length blots with the same results are shown in Fig. S3. (**d**) Immunoblotting of CtTopA and CtHsp60 in cells infected with different *C. trachomatis* strains as indicated. Lysates of cells cultured in aTC-containing medium for 40 h (4–44 hpi) were used. Values are presented as the density of the CtTopA band normalized to the CtHsp60 band from the same sample using ImageJ.

To investigate if endogenous CtTopA in *C. trachomatis* could be recognized by anti-CtTopA or anti-CtTopA_CTD_, we performed an indirect immunofluorescence assay (IFA). HeLa cells were infected with *C. trachomatis* L2/Nt, which expresses WT TopA from the chromosome and harbors a plasmid expressing P*_tet_*-controlled dCas12 and lacking any crRNA (20). In control experiments, we used the L2/*topA*-kd strain, harboring a CRISPRi plasmid with both P*_tet_*-controlled dCas12 and a constitutively transcribed *topA*-specific crRNA that permits specific repression of *topA* when aTC is added. Anti-CtTopA labeled *C. trachomatis* organisms in inclusions of the L2/Nt (Fig. 6a, upper panels), but no signal was detected with anti-CtTopA_CTD_ (not shown). A weaker signal was detected in L2/*topA*-kd inclusions, and such signal was further reduced by adding aTC (Fig. 6a, lower panels), consistent with CRISPRi-mediated repression of *topA* expression (20).

To quantify the expression patterns of endogenous CtTopA, immunoblotting analysis was performed with anti-CtTopA. The levels of CtTopA at 16–42 hpi were assessed because *topA* transcripts were predominantly detected at the mid to late stages of the developmental cycle (17). We observed the appearance of CtTopA in L2/434/Bu (nontransformed WT strain) at 20 hpi and later time points (Fig. 6b and c; Fig. S3). Similar results were obtained with L2/Nt. There were two immunoreactive bands in size around 100 kDa: one corresponding to ~97 kDa CtTopA and the other one at a larger size. A faint band similar to the larger size was detected in mock-infected HeLa cells, and only a single band corresponding to ~97 kDa was observed in purified EBs. Thus, the larger band likely represents non-specific binding to a host cell component, which seemed to be induced by *C. trachomatis* infection. The density of the ~97 kDa band was barely detectable when *C. trachomatis* was exposed to chloramphenicol (an inhibitor of bacterial protein synthesis) (Fig. S4), further indicating that it was derived from *C. trachomatis*.

We next examined CtTopA expression in *C. trachomatis* strains with *topA* knocked down, complemented, or overexpressed. At 44 hpi (the late stage), the CtTopA was detected in L2/Nt but was faintly detected in L2/*topA*-kd (Fig. 6d), in agreement with the IFA data (Fig. 2a). When overexpressing TopA-His6 either in the CRISPRi-complemented strain L2/*topA*-kdcom or the L2/*topA*H6 strain lacking CRISPRi elements (20), a band corresponding in size to ~98 kDa CtTopA was readily detected (Fig. 6d). These results indicate that *C. trachomatis* expresses CtTopA at mid and late stages and that both endogenous CtTopA and ectopically expressed CtTopA from a plasmid (in L2/*topA*-kdcom and L2/*topA*H6) are recognized by anti-CtTopA. It also demonstrates that CRISPRi can effectively repress *topA* transcription and that it, in turn, reduces CtTopA protein levels in *C. trachomatis*.

### Overexpression of mutant TopA with deletion of the SWIB domain slows chlamydial growth

We next asked whether overexpression of TopAΔC affects *C. trachomatis* growth. We transformed the *topAΔC-*encoding plasmid into *C. trachomatis*. The resultant strain, L2/*topAΔC*, was used to infect HeLa cells to assess the impact of TopAΔC overexpression on *Chlamydia* in a wild-type *topA* background. The control vector transformed strain, L2/e.v., was used as a control. The TopAΔC expression was induced by adding aTC/Theo starting at 4 hpi, followed by analysis of *C. trachomatis* growth patterns by measuring inclusion areas and the progeny EB yields.

**Fig 7:**
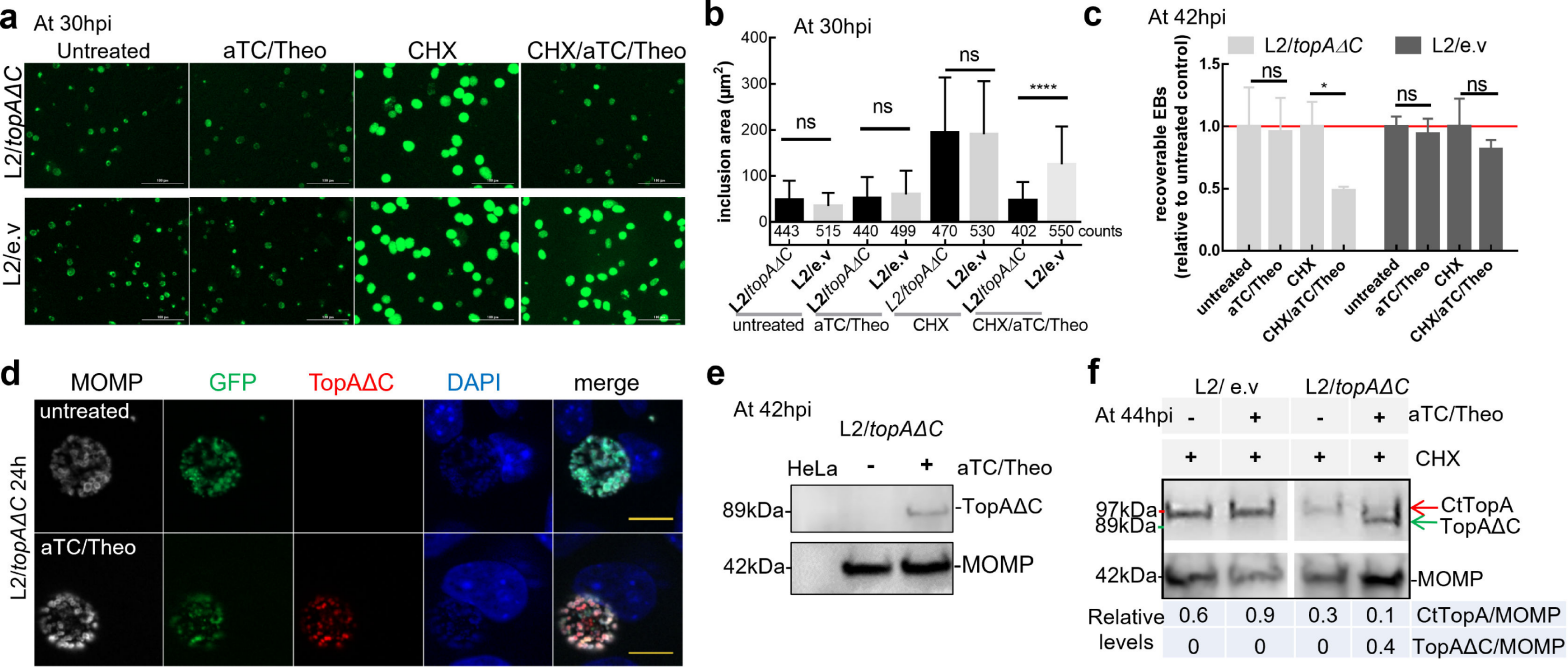
The influence of mutant TopAΔC overexpression on chlamydial growth. *C. trachomatis* L2/*topAΔC* and L2/e.v. at a multiplicity of infection of ~0.4 were used to infect HeLa cells individually. Cells were cultured in the absence or presence of aTC (5 ng/mL)/Theo (15 µg/mL) and/or CHX (1.5 µg/mL) starting at 4 hpi to various times as indicated in each result. (**a**) Live-cell images of *C. trachomatis*-infected cells. Image was taken at 30 hpi under the same exposure conditions. Scale bar = 100 µm. (**b**) Analyzing chlamydial inclusion areas. Inclusion counts were as indicated. Statistical significance was determined by Student’s *t* test, ****P ≤ 0.0001; ns, no significance. (**c**) Numeration of EB yield using infection assay. The infected cells and culture supernatants were collected and used to infect a fresh HeLa cell monolayer to numerate recoverable EBs. The data are presented as the ratio of relative EB yields in the induced cells to those in uninduced cells, which is set at 1 as shown by a red line. The values are presented as the mean ± SD of two independent experiments, each with triplicate. Statistical significance was determined by Student’s *t* test, *P ≤ 0.05. (**d**) Examining inducible expression of TopAΔC in *C. trachomatis* at 24 hpi using IFA. GFP-expressing chlamydial organisms (green) were stained for CtTopA (red; anti-6xHis antibody) and major outer membrane protein (MOMP) (gray; anti-L2 MOMP). Cellular and bacterial DNA was counterstained with 4′,6-diamidino-2-phenylindole dihydrochloride (blue). Scale bar = 10 µm. (**e**) Immunoblot analysis of TopAΔC expression in *C. trachomatis* at 44 hpi with anti- 6xHis and anti-L2 MOMP antibodies. (**f**) Immunoblot analysis of endogenous CtTopA (red arrow) and ectopically expressed TopAΔC (green arrow) in *C. trachomatis* with anti-CtTopA. The protein band density was obtained using ImageJ. The ratios of CtTopA or TopAΔC to those of the MOMP in a representative blot were as indicated. The experiments were repeated three times (Fig. S6).

With normal culture medium, there was no significant difference in chlamydial growth between the –aTC/Theo and +aTC/Theo group in L2/*topAΔC* or L2/e.v. (Fig. 7a through c). We suspected that *C. trachomatis* uses its SWIB domain-containing TopA to aid in its adaptation to host metabolism, given its energy/nutrient dependency on the host cells. Therefore, cycloheximide (CHX) (1.5 µg/mL) was added at 4 hpi to specifically inhibit eukaryotic protein synthesis without direct impact on early events of *Chlamydia* infection (e.g., EB invasion and EB-to-RB transition). In this scenario, host cellular enzymes, processes, nutrient pools, and the immune responses to the infection are altered (35, 36), and *Chlamydia* might outcompete host cells for resources to its own benefit. As expected, the addition of CHX resulted in the formation of larger inclusions and higher EB yields in both L2/*topAΔC* and L2/e.v. (Fig. 7a through c). However, in the presence of CHX/aTC/Theo, *C. trachomatis* L2/*topAΔC* exhibited slow growth, with smaller inclusions and lower EB yields than those cultured in medium with CHX only. In contrast, strain L2/e.v. showed slight growth inhibition after adding CHX/aTC/Theo. Immunodetection with anti-6xHis antibody verified that TopAΔC was stably produced solely in *C. trachomatis* L2/*topAΔC-*infected cells (Fig. 7d and e). The differences in growth between the strains overproducing TopAΔC and the vector control are unlikely due to direct cytotoxicity of CHX, as HeLa cells remain intact at the concentration we used. Rather, the disparities could be, in part, attributed to the altered activity of the mutant TopAΔC.

Since *C. trachomatis* has a chromosomal *topA* in these strain backgrounds, we sought to determine whether the endogenous CtTopA was stably produced. Immunoblot analysis was performed with the lysates of *C. trachomatis* L2/*topAΔC* or L2/e.v.-infected cells grown in medium with CHX or CHX/aTC/Theo. We simultaneously probed for endogenous CtTopA (~97 kDa) and the ectopically expressed TopAΔC (~89 kDa) with anti-CtTopA (Fig. 7f; Fig. S6). In the presence of CHX, a single band of endogenous CtTopA was detected in L2/*topAΔC* and L2/e.v. at 44 hpi. Upon addition of CHX/aTC/Theo, the levels of CtTopA in L2/e.v. were almost unchanged, while TopAΔC production was induced, and stable expression of CtTopA was also detected in L2/*topAΔC*. These results indicate the co-existence of CtTopA and the TopAΔC proteins in L2/*topAΔC*-infected cells, providing a possible explanation of slow growth.

## DISCUSSION

Here, we characterized the function of chlamydial SWIB-domain containing CtTopA using a combined approach of genetics and biochemistry. Our work indicates that *C. trachomatis* naturally produces a unique TopA ortholog, which exerts its role as a critical regulator for DNA relaxation during mid and late stages of chlamydial development and is required for *C. trachomatis* to productively multiply in host cells.

### Bacterial TopA C-terminal domains display sequence and structure diversity

Bacterial TopAs consist of structural elements at the C-termini involved in DNA binding and relaxation (2, 25). Two prototypes of C-terminal motifs in TopAs have been well defined. The Topo_C_ZnRpt (with 4-cysteine for Zn coordination) initially identified in EcTopA (37, 38) was found in the phyla of Proteobacteria and Firmicutes. The Topo_C_Rpt (without cysteine) has been identified in *M. tuberculosis* and the phyla of Actinobacteria (39). Although much remains to be studied, variations in the combination of these two motifs in specific organisms have been increasingly noted (2, 25). For example, both Topo_C_ZnRpt and Topo_C_Rpt can be found in the TopAs from Proteobacteria *Rickettsia bellii* and *Caulobacter crescentus*. Our bioinformatic inspection confirms that *Chlamydia* spp. encode a eukaryotic SWIB domain-containing TopA, unlike other characterized TopA orthologs. The acquisition of protein domains with phylogenetic signatures suggesting eukaryotic origin by *Chlamydia* is perhaps unsurprising as *Chlamydia* is an obligate intracellular bacterial parasite that has evolved within eukaryotic host cells (12, 40, 41). The amino acid sequence of the SWIB domain in CtTopA appears to fold as a discrete protein structure within the overall CtTopA structure as determined by AlphaFold modeling (Fig. 1). Notably, an apparently unstructured region directly precedes the SWIB domain, suggesting potential flexibility in the protein that may allow this domain to dynamically interact with other proteins or regions of DNA. Further work is required to test this as well as the precise function of the SWIB domain. Nonetheless, the presence of a SWIB domain in chlamydial TopA distinguishes this ortholog from the known domain organization of other TopAs. This highlights a new paradigm of potential variations within the TopA family likely driven by pathogen-specific intracellular adaptations.

### Comparison between CtTopA and EcTopA activity

Comparison of the DNA relaxation efficiency of CtTopA with EcTopA provides useful clues to the mechanisms and enzyme kinetics of these evolutionary divergent TopAs. We found that the activity of DNA relaxation of CtTopA is lower than that of *E. coli* TopA, and CtTopA was unable to complement *E. coli topA* mutants as effectively as the *E. coli* ortholog. The weakened ability of CtTopA could have several explanations. First, the C-terminal zinc ribbon-like domains (D8 and D9) of EcTopA (38) are not found in CtTopA, which instead has the SWIB domain (Fig. 1). The D8 and D9 of EcTopA have been shown to bind to ssDNA with high affinity (42) and have been proposed to play a vital role in the relaxation activity of EcTopA (28). The relaxation activity of CtTopA would be less efficient if the SWIB domain of CtTopA does not bind the ssDNA region of negatively supercoiled DNA with an affinity similar to that of zinc ribbon-like domains. Second, the C-terminal zinc finger and zinc ribbon domains of EcTopA also participate in specific protein-protein interactions between EcTopA and the RNA polymerase (35). Because of the likely lack of identical protein-protein interactions, CtTopA may not be as effective as EcTopA in removing transcription-driven negative supercoils during transcription elongation and preventing R-loop formation (36, 43–45), thus limiting the degree of complementation of *topA* mutation in *E. coli*. Finally, the degree of complementation by the CtTopA clone in *E. coli* may also be influenced by the plasmid copy number variation (46). The AT-rich *C. trachomatis* genome vs the high GC content in *E. coli* genome is unlikely the reason for the lower efficiency of CtTopA relaxation activity. The only DNA sequence preference shared by EcTopA, MtTopA, and all other bacterial TopAs characterized to date is the presence of a cytosine at position −4 (upstream) of the site cleaved by the enzyme to form the covalent intermediate (47). A binding pocket for the cytosine base is formed by EcTopA residues R169, R173, and Y177, all of which are conserved in bacterial TopAs’ sequences, including in CtTopA. It is possible that CtTopA uses a similar binding pocket formed by these three residues to bind a cytosine in a step to position the active site tyrosine nucleophile close to the scissile phosphate during catalysis. We observed that overproduction of TopAΔC in *E. coli* could complement the growth defect in *E. coli* AS17 at the non-permissive temperature (42°C) (Fig. 5), consistent with the idea that some mechanistic conservations are determined by the N-terminal domain of TopAs. On the other hand, the ssDNA binding repeats in the TopA C-terminal domains do contribute to substrate binding and processivity during relaxation of negatively supercoiled DNA (25, 48, 49). Since CtTopA has only three of the ssDNA binding Topo_C_ZnRpt repeats, it may not be as efficient in catalysis as EcTopA, which has five ssDNA binding Topo_C_ZnRpt repeats. The findings that TopAΔC overexpression resulted in reduced complementation ability compared to the CtTopA in *E. coli* (Fig. 5) support the hypothesis that the SWIB domain contributes to the relaxation activity of CtTopA.

### SWIB domain-containing CtTopA contributes to the chlamydial developmental cycle

Our results substantiate previous findings that the CtTopA plays a regulatory role in the *C. trachomatis* developmental cycle (20). Endogenous CtTopA protein was produced in a temporal manner (Fig. 6) consistent with previously reported transcription analyses (16). Interestingly, overexpression of TopAΔC in *C. trachomatis* did not impact growth in normal medium but did result in slow chlamydial growth, specifically when host protein synthesis was inhibited (Fig. 7a through c). These results suggest that *Chlamydia* needs intact CtTopA in response to metabolic changes induced by inhibition of host cell protein synthesis. In particular, *C. trachomatis* growth is contingent on the availability of nutrients (nucleotides, lipids, and amino acids) in the host cells and can dictate metabolic flexibility to remodel the cell interior to its own advantage. Such complex host cell-pathogen interactions can be a source of various stresses for *Chlamydia*. Many types of stresses have been associated with supercoiling changes in bacteria (50). Perhaps it is crucial that CtTopA exerts its DNA relaxation function as part of physiological tasks to balance DNA supercoiling, allowing *Chlamydia* to survive in a hostile metabolic environment within the host cell.

Despite the paucity of understanding of the role of the SWIB domain in bacteria, the TopA C-terminal domains can perform various roles, including binding to DNA/RNA, catalyzing reactions, or interacting with specific protein partners depending on the bacterial species (2, 25). The eukaryotic SWIB domains, sharing structural similarities with MDM2 (22, 23), could, by binding to transcription factors and potentially DNA, enable the protein complex to target specific regions of chromatin to remodel gene regulation. Whether the chlamydial SWIB domain is involved in specific protein-protein interactions with different proteins affecting gene expression remains to be determined to clarify how exactly the SWIB domain affects CtTopA activity in *C. trachomatis*. Nevertheless, there might be multiple explanations for the slow growth caused by TopAΔC overexpression. For example, due to the absence of the SWIB domain, TopAΔC could compete with endogenous CtTopA to bind to chromosomal DNA and be deficient in protein-protein interactions or inefficient in catalytic activity required for optimal cellular processes. Further investigation is warranted to more completely understand the relationship between domain composition and function of CtTopA.

## MATERIALS AND METHODS

### Reagents

Oligonucleotides and primers were synthesized by Integrated DNA Technologies (Coralville, IA, USA). Restriction enzymes, T4 DNA ligase, and RNase inhibitors were purchased from New England Biolabs (Ipswich, MA, USA). Cycloheximide, antibiotics, and deoxynucleotide were purchased from Sigma-Aldrich or ThermoFisher Scientific.

### Bioinformatics analysis

The amino acid sequences of CtTopA (CLT0011) and its counterparts in *E. coli*, *M. tuberculosis*, *H. pylori*, *P. aeruginosa*, and *N. gonorrhoeae* were obtained from the UniProt Knowledgebase (UniProtKB) (https://www.uniprot.org/). ClustalW multiple sequence alignment was conducted with Matrix BLOSUM62. Domains of CtTopA and its structural model were predicted by InterPro and AlphaFold, respectively. The amino acid sequence of CtTopA protein was used in protein-protein BLAST (BLASTp) at National Center for Biotechnology Information (NCBI) (https://blast.ncbi.nlm.nih.gov/) searches against the non-redundant standard database corresponding to the *Chlamydiae*/Verrucomicrobia group (taxid:1783257). A maximum of 5,000 aligned sequences was selected for display, using an *E*-value of 0.05 as threshold and the BLOSUM62 matrix. NCBI MSA Viewer 1.25.0 was used to visualize amino acid alignment of the SWIB-domain regions from the top 500 homologs of CtTopA.

### Plasmids and expression of recombinant TopAs in *E. coli*

The strains and plasmids used in this study are listed in Table S2. The full-length coding sequence of CtTopA (857 amino acid residues) optimized for expression in *E. coli* was custom synthesized by Gene Universal (Delaware, USA) and inserted into vector pET28a(+) for the expression of recombinant CtTopA with N-terminal 6xHis tag. The resulting pET-CtTopA plasmid was transformed into *E. coli* strain BL21(DE3). Transformants were grown in LB (Miller) broth with 50 µg/mL kanamycin at 37°C for overnight culture. The next day, the overnight cultures were diluted 1:100 in LB with 50 µg/mL kanamycin and grown until OD_600_ reached 0.4. Recombinant protein expression from the T7 promoter was induced with the addition of 1 mM IPTG. The cells were harvested after additional growth at 37°C for 4 h. Similarly, plasmid expressing EcTopA (28) or MtTopA (29) (Table S2) was used for the expression of these recombinant topoisomerases in BL21 STAR (DE3) strain (Invitrogen) and C41(DE3) (Lucigen), respectively.

The pBOMB4-derived shuttle plasmid pBOMBE contained dual control of anhydrotetracycline-inducible promoter P*_tet_* and a theophylline-dependent riboswitch. This allows us to tightly control the expression of the gene of interest at the transcription and translation levels to achieve robust and specific gene repression. To construct a mutant allele with a deletion of the SWIB region, the DNA fragment containing a 2,331 bp *topA* coding region lacking the 3′ SWIB region and 6xHis (including a stop codon) was cloned into pBOMBE, resulting in pBOMBE*topAΔC*.

### Purification of recombinant CtTopA, EcTopA, and MtTopA

EcTopA and MtTopA were purified as previously described (28, 29). For purification of CtTopA, the pelleted bacterial cells were resuspended in a buffer containing 50 mM sodium phosphate, pH 7.4, 0.3 M NaCl, and 20 mM imidazole. After the addition of 1 mg/mL lysozyme, the cells were left on ice for 1 h before three cycles of freeze-thaw to lyse the cells. The soluble lysate obtained after centrifugation at 32,000 rpm for 2 h was mixed with Ni-NTA agarose (from Invitrogen, Thermo Fisher) and packed into a column. After washing, the protein was eluted with a buffer containing 50 mM sodium phosphate, pH 7.4, 0.3 M NaCl, and 250 mM imidazole. Protein concentration was determined with the Bradford assay.

### *In vitro* assay of topoisomerase relaxation activity

The relaxation activity assay was conducted in 20 µL of 10 mM Tris-HCl, pH 8.0, 50 mM NaCl, 0.1 mg/mL gelatin, and 2 mM MgCl_2_ with 0.3 µg of negatively supercoiled pBAD/thio plasmid DNA as substrate. Following the addition of topoisomerase, the reactions were incubated at 37°C for the length of time indicated in the results and stopped by the addition of 4 µL of stop solution (50 mM EDTA, 50% glycerol, and 0.5% [vol/vol] bromophenol blue). The supercoiled DNA substrate and relaxed DNA products were separated by electrophoresis in a 1% agarose gel with TAE (40 mM Tris-acetate [pH 8.0] and 2 mM EDTA) buffer. Following staining with 1 µg/mL ethidium bromide, the gel was de-stained with deionized water and then photographed with UV light and the Alpha Imager Mini. Percent relaxation was determined as previously described (30). The migration distance of supercoiled DNA, fully relaxed DNA, and partially relaxed DNA bands was identified using AlphaViewer. The weighted distance of PR bands for each lane was calculated from the data obtained. The percentage of relaxation was calculated with the formula (SC − PR)/(SC − FR) × 100.

### Complementation of *topA* mutations in *E. coli*

The complementation assay was performed as described by Annamalai and Tse-Dinh (51). Briefly, the *E. coli-C. trachomatis* shuttle plasmids (pBOMBLs-*topA*H and vector control pBOMBLs) were transformed into *E. coli* VS111-K2 with *topA* mutation (Table S2). The transformants grown in LB broth with 50 µg/mL spectinomycin and 30 µg/mL chloramphenicol at 37°C overnight were diluted to OD_600_ = 0.1 prior to serial 10-fold dilutions. The dilutions were spotted on LB agar plates and incubated at 30°C, 37°C, and 42°C for 18 h before imaging. For the growth curve, the overnight culture was diluted in fresh LB at a ratio of 1:100, cultured in LB containing aTC or not at 37°C, and sampled to measure the optical density at 600 nm (OD_600_) every 2 h. The absorbance values were plotted against the growth time.

The pET-CtTopA plasmid was transformed into the *E. coli* AS17 strain (Table S2), which has a temperature-sensitive *topA* mutation and requires complementation for growth at 42°C (29, 34). The pLIC-EcTOP plasmid (28) expressing His-tagged *E. coli* TopA controlled by the T7 promoter was used as a positive control for comparison, along with an empty vector as a negative control. Similarly, the pBOMBE*topAΔC* and vector control pBOMBE were introduced into AS17. Individual AS17 transformants were isolated at 30°C as biological replicates. The cultures grown in LB broth containing proper antibiotics at 30°C overnight were either streaked on LB agar plates or diluted with LB for an OD_600_ value to equal 0.1. Tenfold serial dilutions were prepared for spotting 5 µL of each dilution onto LB agar plates. The plates were photographed following incubation at 30°C for 36 h or 42°C for 18 h.

### Cell culture and *C. trachomatis* infection

HeLa 229 cells (human cervical epithelial carcinoma cells; ATCC CCL-2) were cultured in RPMI 1640 medium (Gibco) containing 5% heat-inactivated fetal bovine serum (Sigma-Aldrich), gentamicin 20 µg/mL, and L-glutamine (2 mM) (RPMI 1640-10) at 37°C in an incubator with 5% CO_2_. Cells were confirmed to be *Mycoplasma* negative by PCR as described previously (52). To propagate and prepare the large amounts of EBs, HeLa cells grown in T175 flasks or 6-well culture plates were infected with *C. trachomatis* (Table S2) and cultured in RPMI 1640-10 at 37°C for 45 hpi. For transformed strains, the medium was supplied with spectinomycin (500 µg/mL). Cells were harvested for EB purification as described previously (53). The purified EB pellet was resuspended in sucrose-phosphate-glutamic acid buffer (10 mM sodium phosphate, 220 mM sucrose, and 0.50 mM L-glutamic acid). The EB aliquots were stored at −80°C until use. Serial dilutions of EBs were used to determine the titers of recoverable EBs in 96-well plates as inclusion-forming units. For growth phenotypic analysis, *C. trachomatis* EBs were used to infect cells grown in 96-well plates (catalog #655090, Greiner) with a dose that results in ~30% –40% of cells being infected. After centrifugation with a Beckman Coulter model Allegra X-12R centrifuge at 1,600 × *g* for 45 min at 37°C, fresh medium was added to the infected cells and incubated at 37°C for various time periods as indicated in each experimental result. For comparison, different strains were infected side by side in the same culture plate with a setup of at least triplicate wells per condition.

### CtTopA antibody production

Purified full-length His6-CtTopA was used to produce polyclonal antibody against chlamydial TopA in mice as described previously (54). Briefly, 50 µg of recombinant TopA emulsified in equal volumes of complete Freund’s Adjuvant was intraperitoneally injected into a mouse. Two weeks later, the same amount of TopA antigen, emulsified in incomplete Freund’s Adjuvant, was similarly injected twice at an interval of 2 weeks. Sera were collected 2 weeks after the final booster injection. The synthesized peptides corresponding to amino acids 737–756 and 843–857 of CtTopA were used to produce antibody in rabbit (Pacific Immunology). The final serum was purified by an affinity column, dialyzed against PBS with 40% glycerol, and the aliquots were stored at −20°C prior to use.

### Immunofluorescence microscopy

Live cell images of *C. trachomatis*-infected HeLa were obtained at various time points as described in each result using the Cytation 1 multimode reader (BioTek Instruments, Winooski, VT, USA). For IFA, *C. trachomatis*-infected HeLa cells cultured for 24 or 42 hpi were fixed with 4% formaldehyde for 15 min and permeabilized by using 0.1% Triton X-100 for 15 min, followed by immunostaining with proper antibody overnight at 4°C. After extensively washing, cells were then incubated with Alexa Fluor 568-conjugated secondary antibody (1:200; Molecular Probes) for 45 min at 37°C and counterstained with 4′,6-diamidino-2-phenylindole dihydrochloride. Images were taken by a Zeiss Axiovert.Z2 with Apotome 2 with a 100× objective.

### Infection assay to enumerate infectious *C. trachomatis* EB

*C. trachomatis*-infected cells and the culture medium were frozen at −80°C, thawed once, scraped into the medium, serially diluted, and then used to infect a fresh monolayer of HeLa cells in 96-well plates. The cells were cultured in RPMI 1640-10 with 500 µg/mL spectinomycin at 37°C for 40 h, fixed with 4% paraformaldehyde, and permeabilized with 0.1% Triton X-100. Antibody specifically against the major outer membrane protein (MOMP) of *C. trachomatis* LGV L2 was used for immunostaining (55). Images were taken, and the inclusion numbers in triplicate wells were counted and converted to inclusion-forming unit per milliliter for comparison.

### Immunoblots

For western blot analysis, *C. trachomatis*-infected cells in 12-well culture plates were lysed directly in 8 M urea buffer containing 10 mM Tris-HCl (pH 8.0), 0.1% SDS, and 2.5% β-mercaptoethanol. The protein content was determined by a bicinchoninic acid protein assay kit (Thermo Fisher). Cellular lysate was prepared from each sample, and an equal amount of protein was loaded into a single lane of the 4%–15% SDS-polyacrylamide gel (BioRad). After electrophoresis and transfer to a polyvinylidene difluoride membrane (Millipore), the membrane was incubated with the following antibodies individually: anti-CtTopA (1:2,000), anti-CtTopA_CTD_ (1:2,000), anti-Hsp60 (1:500) (56), anti-L2 MOMP, anti-His6 antibody (Abcam), or the loading control host GAPDH (1:2,000) (MilliporeSigma), followed by incubation with the HRP-conjugated secondary antibody. For dot blot analysis, the serial 10-fold dilutions of *E. coli* cultures were spotted and grown on the agar plate at 30°C for 48 h. The bacteria were directly transferred to a nitrocellulose membrane and lysed by chloroform. After blocking and washing steps, the membrane was incubated with anti-CtTopA (1:2,000 dilution), followed by incubation with the HRP-conjugated secondary antibody. All the blots were imaged on an Azure c600 imaging system. The relative density of a given protein band is evaluated across its respective row by ImageJ.

### Statistical analysis

Data analyses were performed using Prism (version 10; GraphPad, San Diego, CA, USA). *P* values of < 0.05 were considered statistically significant. The details were indicated in each result.

## SUPPLEMENTAL MATERIAL

Figures S1 to S6, and Tables S1 and S2. jb.00190-25-s0001.pdf Fig. S1: Protein sequence alignment. Fig. S2: Time course of relaxation assay. Fig. S3: Full-length blots (time course). Fig. S4: Blots showing inhibition of CtTopA by Cm. Fig. S5: Dot blot S6 co-presence of CtTopA and mutant TopAΔC. Fig. S6: The immunoblots from three independent experiments.Table S1: Alignment of the C-terminal amino acid residues from homologues of CtTopA in *Chlamydia spp*. Table S2: Strains and plasmids used in this study.

ASM does not own the copyrights to Supplemental Material that may be linked to, or accessed through, an article. The authors have granted ASM a non-exclusive, world-wide license to publish the Supplemental Material files. Please contact the corresponding author directly for reuse.
