# Supplementary material for: The SWIB domain-containing DNA topoisomerase I of Chlamydia trachomatis mediates DNA relaxation
Source: J Bacteriol. 2025 Aug 12;207(9):e00190-25. doi: 10.1128/jb.00190-25 (PMC12445093; doi:10.1128/jb.00190-25)
Supplement: Figures S1 to S6, and Tables S1 and S2. — Fig. S1: Protein sequence alignment. Fig. S2: Time course of relaxation assay. Fig. S3: Full-length blots (time course). Fig. S4: Blots showing inhibition of CtTopA by Cm. Fig. S5: Dot blot S6 co-presence of CtTopA and mutant TopAΔC. Fig. S6: The immunoblots from three independent experiments.Table S1: Alignment of the C-terminal amino acid residues from homologues of CtTopA in Chlamydia spp. Table S2: Strains and plasmids used in this study. [file jb.00190-25-s0001.pdf]

Supplemental data

The SWIB domain-containing *Chlamydia trachomatis* DNA topoisomerase I mediates DNA relaxation

Figures S1-S6

Table S1 and S2

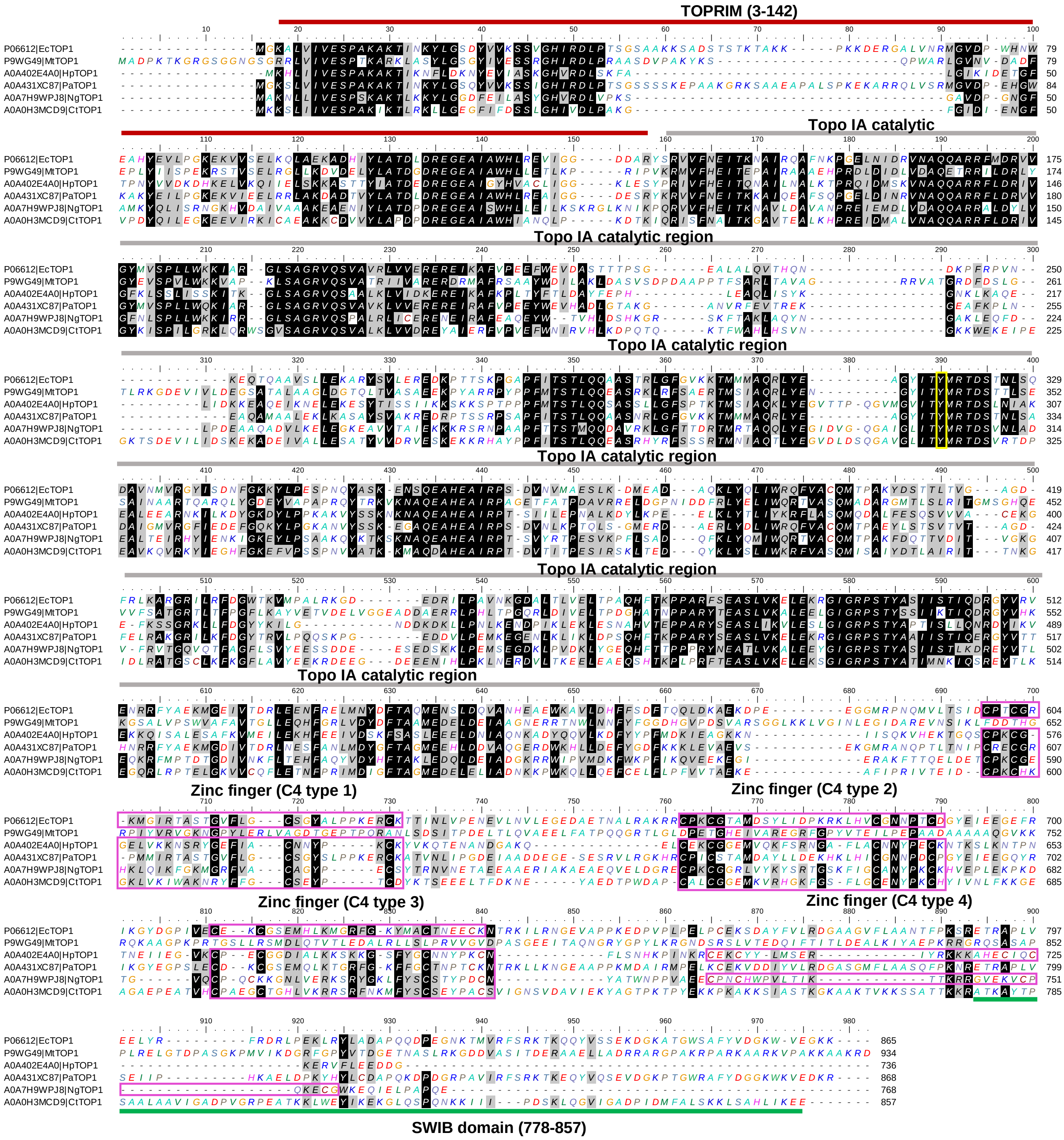

Figure S1 Amino acid seqence alignment of TopAs from *E. coli*, *M. tuberculosis*, *H. pylori*, *P. aeruginosa*, *N. gonorrhea*, *and C. trachomatis* using ClustalW with BLOSUM62 matrix. Conserved TOPRIM and catalytic regions are indicated by the red and gray lines, respectively. Residues corresponding to the conserved active site of tyrosine (Y) are boxed (yellow). The region corresponding to conserved 4C zinc fingers are framed. The SWIB domain at the CTD of CtTopA is underlined (green). Also see Figure 1.

a

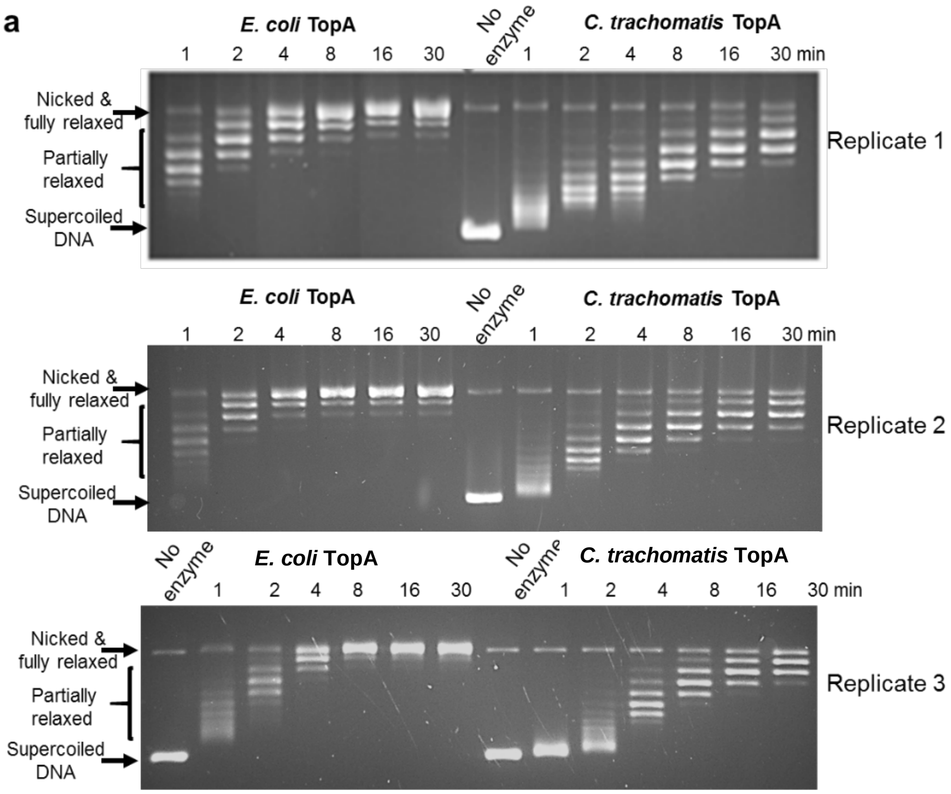

b

% DNA relaxation

Time (min)	EcTopA			CtTopA		
	Rep1	Rep2	Rep3	Rep1	Rep2	Rep3
0	0.	0.	0.	0.	0.	0.
1	65.	63.	77.	24.	16.	7.
2	81.	87.	72.	44.	44.	17.
4	96.	96.	93.	49.	62.	52.
8	91.	97.	100.	64.	73.	71.
16	97.	97.	99.	71.	80.	82.
30	97.	97.	100.	78.	85.	88.

Figure S2. Time course of DNA relaxation of CtTopA and EcTopA *in vitro*. EcTopA or CtTopA (25 nM) was incubated with 0.3 μg negatively supercoiled DNA for different times (0-30 min), followed by agarose gel electrophoresis. (**a**) The gels of three independent experiments. (**b**) Quantification of DNA relaxation based on (**a**). The percent of relaxation was determined by dividing the distance between the negatively supercoiled band (SC); and the weighted center of the partially relaxed band (PR); by the distance between the supercoiled band (SC); and the fully relaxed band (FR). (Formula: percent relaxation = (SC-PR)/(SC-FR)*100. (also see Fig. 2d).

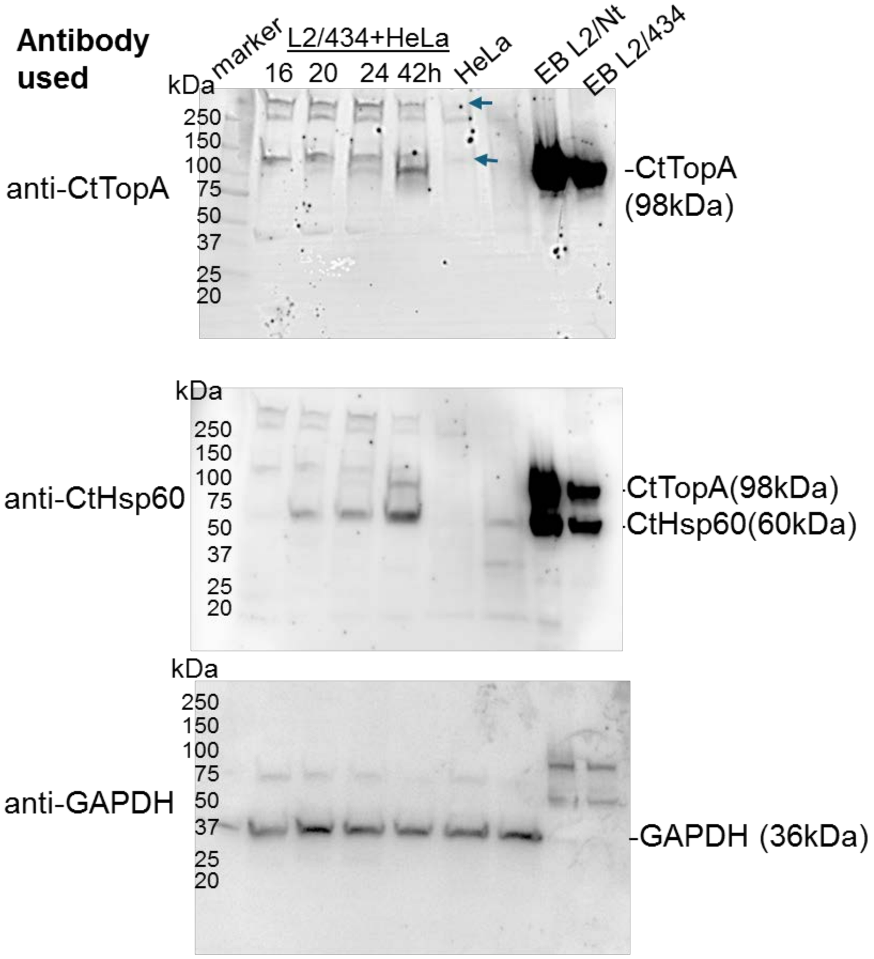

Figure S3. The full-length blots showing analysis of endogenous chlamydial CtTopA levels. Lysates of *C. trachomatis* infected HeLa cells sampled at 16, 20, 24, and 42 h pi. *C. trachomatis* Hsp60 (CtHsp60) and the host cell glyceraldehyde 3-phosphate dehydrogenase (GAPDH) were used as controls. The equal amounts of proteins for each sample were loaded. The membrane was used for immunoblotting with anti-CtTopA (upper panel) and then anti-CtHsp60 (middle panel). After washing with strip buffer, the membrane was reprobed for GAPDH (low panel). The same data are shown in Figure 5b. Note: several bands cross reacted with anti-CtTopA. Two bands, as indicated by arrows, corresponding to >250kDa or corresponding to >100kDa, appeared to be induced in *C. trachomatis* infection.

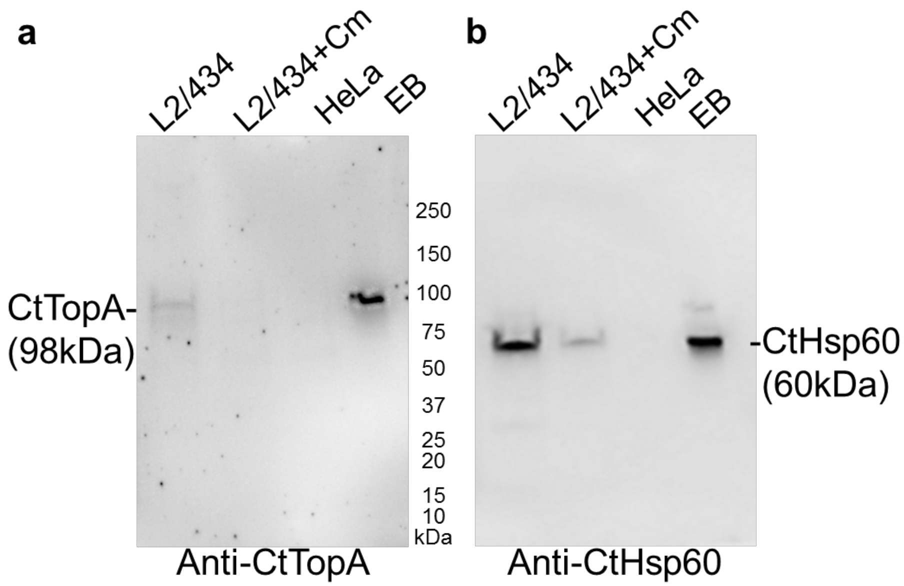

Figure S4. Analyzing influence of chloramphenicol (Cm) on CtTopA and CtHsp60 expression. using Western blot. HeLa cell infected with *C. trachomatis* L2/434/Bu were exposed to Cm at 60 μg/mL starting from 18h pi for 30 hrs (to 48h pi) or not. Mock infected HeLa cells and purified EBs were used as negative and positive controls, respectively. Cells were harvested at 48h pi and lysed in in 8 M urea buffer containing 10 mM Tris-HCl (pH 8.0), 0.1% SDS, and 2.5% β-mercaptoethanol. An equal amount of total protein for each sample as determined by BCA assay was used for immunoblotting with anti-CtTopA (**a**) or anti-CtHsp60 (**b**).

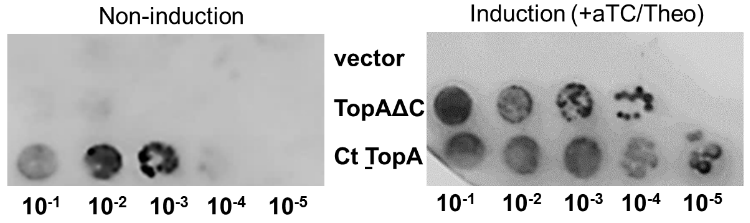

Figure S5. Dot blot analysis of CtTopA and TopAΔC expression in *E. coli* AS17. The serial 10-fold dilutions of *E. coli* AS17 cultures with CtTopA, TopAΔC, or vector control were spotted and grown on the agar plate at 42˚C for 36 hrs in the absence (left) or presence (right) of aTC (at 5ng/mL)/Theo (at 15 μg/mL). The bacteria were transferred to a nitrocellulose membrane and lysed by chloroform. The proteins were probed with anti-CtTopA. Note: the presence of TopAΔC at approximately the same level as CtTopA after induction by adding aTC/Theo.

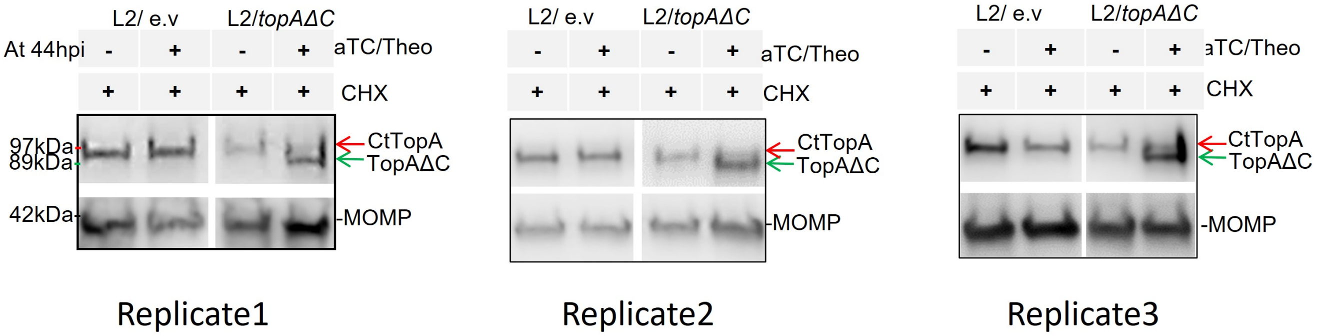

Figure S6. The immunoblots from three independent experiments show that the stable productions of endogenous CtTopA (read arrows) were co-present with the ectopically expressed mutant TopAΔC (green arrows) in *C. trachomatis* L2/*topAΔC* at 42h pi. The expression of TopAΔC from the plasmid was induced by adding aTC (at 5ng/mL)/Theo (at 15 μg/mL) starting at 4 h pi. Also see Fig. 7f.

Table S1 Alignment of the C-terminal amino acid residues from homologues of CtTopA in *Chlamydia* spp.

NCBI Multiple Sequence Alignment Viewer, Version 1.25.2

Sequence ID	Start	Alignment (750 – 856)	End	Organism
CTL 0011A0A0H3MCD9	1	KPKAKKSIASTKGKAAKTVKKSSATTKKRATKAYTPSAALAAVIGADPVGRPEATKKLWEYIKEKGLQSPQNKKIIIPDSKLQGVIGADPIDMFALSKKLSAHLIKE	857	
WP_009873260.1 (+)	1		857	Chlamydia trachomatis
WP_012263525.1 (+)	1		857	Chlamydia trachomatis
ADI51319.1 (+)	14		870	Chlamydia trachomatis ...
WP_010725288.1 (+)	1		857	Chlamydia trachomatis
WP_015506113.1 (+)	1		857	Chlamydia trachomatis
WP_009872013.1 (+)	1	T	857	Chlamydia trachomatis
WP_015506487.1 (+)	1		857	Chlamydia trachomatis
WP_069568511.1 (+)	1		857	Chlamydia trachomatis
WP_322631943.1 (+)	1		857	Chlamydia trachomatis
WP_009872811.1 (+)	1	V	857	Chlamydia trachomatis
WP_123640163.1 (+)	1	V	857	Chlamydia trachomatis
WP_100102813.1 (+)	1	V	857	Chlamydia trachomatis
WP_020966639.1 (+)	1	V	857	Chlamydia trachomatis
WP_020967061.1 (+)	1	V	857	Chlamydia trachomatis
WP_010229101.1 (+)	1	TASK GKATKG ASTT K TV N K AT S P I F I A Q N RV V MKH D IQ T Q	865	Chlamydia muridarum
WP_080141968.1 (+)	1	A T AVS K GKTTKA KDLIA T KAP S SL A DR SN R L D KL SE A N S	856	Chlamydia suis
WP_082192381.1 (+)	1	A T AVS KEA TK A SSKT K TRK P S SH A DR SS R L D KL SE A N G	868	Chlamydia suis
WP_080125656.1 (+)	1	A T AVS KEA TK A SSKE K KAP S SH A DR SS R L D KL SE A N G	879	Chlamydia suis
WP_080124481.1 (+)	1	A T AVS KEA TK A SSKE K KAP S SL A DR SN R L D KL L S A N G	889	Chlamydia suis
MDD7385427.1 (+)	1	A T AVS K TT A SSKT K TRK P S SL A DR SN R L D KL L SE A N G	868	Chlamydia suis
WP_082196865.1 (+)	1	A T VVS K GKTTKA KDLIA T KAP S SL A DR SN R L D KL S A N S	856	Chlamydia suis
WP_080122531.1 (+)	1	A T VVS K GKTTKA KDLIA T KAP S SL A DR SN R L D KL S A N S	856	Chlamydia suis
WP_080142412.1 (+)	1	A T VVS K GKTTKA KDLIA T KAP S SL A DR SN R L D KL S A N S	856	Chlamydia suis
WP_087878153.1 (+)	1	A T AVS KEA TK A SSKE K KAP S SH A DR SS R L D KL SE A N G	879	Chlamydia suis
WP_219685801.1 (+)	1	A T VVS K GKTTKA KDLIA T KAP S SL A DR SN R L D KL S A N S	856	Chlamydia suis
WP_080127242.1 (+)	1	A T AVS KEA TK A SSKT K TRK P S SH A DR SS R L D KL SE A N G	868	Chlamydia suis
WP_080128527.1 (+)	1	A T AVS KEA TK A SSKDP A T KAP S SL A DR SN R L D KL SE A N G	867	Chlamydia suis
MCI5641838.1 (+)	1	A T VVS K GKTTKA KDLIA T KAP S SL A DR SN R L D KL S A N S	856	Chlamydia suis
WP_080132675.1 (+)	1	A T AVS KEATTAKK DPT K TRK P S SL A DR SN R L D KL L SE A N G	868	Chlamydia suis
WP_080132172.1 (+)	1	G TT AVS KEA TT A KDP A P KAP S SL A DR SN R L D KQ SE A N G	867	Chlamydia suis
WP_080123174.1 (+)	1	A T AAS K TT A SSKE A P KAP S SL A DR SN R L D KL L S A N G	867	Chlamydia suis
WP_080129129.1 (+)	1	G TT AVS KEA TT A KDP A T KAP S SH A DR SN R L D KL L SE A N G	867	Chlamydia suis
WP_080129701.1 (+)	1	A T VVS K GKTTKA KDPIA T KAP S SL A DR SN R L D KL S A N S	856	Chlamydia suis
WP_080126757.1 (+)	1	EA T AVS KET TK A SSKT K TRK P S SL A DR SS R L D KL L SE A N G	879	Chlamydia suis
WP_087877914.1 (+)	1	G TT AVS KEA TT A KDP A T KAP S SH A DR SN R L D KL L S A N G	867	Chlamydia suis
WP_080129495.1 (+)	1	A T AAS K TT A SSKE A P KAP S SL A DR SS R L D KL L SE A N G	867	Chlamydia suis
WP_080123916.1 (+)	1	A T AAS KEA TK A SSKDP A T KAP S SL A DR SN R L D KL SE A N G	867	Chlamydia suis
WP_080126096.1 (+)	1	G TT AVS KEA TT AK DPTAKTT KAP S SL A DR SN R L D KL L SE A N G	868	Chlamydia suis
WP_080140929.1 (+)	1	A T AAS K TT A SSKE A P KAP S SL A DR SS R L D KL S A N G	867	Chlamydia suis
WP_080133153.1 (+)	1	EA T AVS KEA TK A SSKDP A T KAP S SL A DR SN R L D KL SE A N G	878	Chlamydia suis
WP_291894236.1 (+)	1	K VASTK ES IT P LS TVPT M P L SL I A AN LD RV V K EE N G	856	Chlamydia sp.
WP_080141266.1 (+)	1	A T AAS K TT A SSKE A P KAP S SL A DR SS R L D KL L SE A N G	867	Chlamydia suis
WP_080121617.1 (+)	1	G TT AVS KEA TT AK DPTAKTT KAP S SL A DR SN R L D KL L SE A N G	868	Chlamydia suis
WP_080128743.1 (+)	1	A T AVS KEA TK A SSKE K KTK VS AYTPS ALA SL A DR SS R L D KL L S A N G	911	Chlamydia suis
WP_057267663.1 (+)	1		649	Chlamydia trachomatis
CRH26064.1 (+)	1		644	Chlamydia trachomatis
WP_006343649.1 (+)	1	TTG KATTKSSSKTTKTTAKKKKSEGKTP TL PQ LM PE G V Q DHN E MLL E FEAI PE V Q P L NQ F S	862	Chlamydia
WP_014946333.1 (+)	1	TTG KATTKSSSKTTKTTAKKKKSEG TP TL PQ LM PE G V Q DHN E MLL E FEAI PE V Q P L NQ F S	862	Chlamydia psittaci
WP_014945663.1 (+)	1	TTG KATTKSSSKTTKTTAKKKKSEGKTP TL PQ LM PE G V Q DHN E MLL E FEAI PE V Q P L NR F S	862	Chlamydia psittaci
WP_014946975.1 (+)	1	TTG KATTKSSSKTTKTTAKKKKSEGKTP TL PQ LM PE G V Q DHN E MLL E FEAI PE V Q P L NQ F S	862	Chlamydia psittaci
WP_016981692.1 (+)	1	TTG KATTKSSSKTTKTTAKKKKSEGKTP TL PQ LM PE G V Q DHN E MLL E FEAI PE V Q P L NQ F S	862	Chlamydia psittaci
WP_014944633.1 (+)	1	TTG KATTKSSSKTTKTTAKKKKSEGKTP TL PQ LM PE G V Q DHN E MLL E FEAI PE V Q P L NQ F S	862	Chlamydia psittaci
WP_014945419.1 (+)	1	TTG KATTKSSSKTTKTTAKKKKSEGKTP TL PQ LM PE G V Q DHN E MLL E FEAI PE V Q P L NQ F S	862	Chlamydia psittaci
WP_127345299.1 (+)	1	TTG KATTKSSSKTTKTTAKKKKSEGKTP TL PQ LM PE G V Q DHN E MLL E FEAI PE V Q P L NQ F S	862	Chlamydia psittaci
WP_016966554.1 (+)	1	TTG KATTKSSSKTTKTTAKKKKSEGKTP TL PQ LM PE G V Q DHN E MLL K FEAI PE V Q P L NQ F S	862	Chlamydia psittaci
WP_011457582.1 (+)	1	TTS KSTTKASSKTTKTTAKKKKSA K GSLLI PQ QM NE A G I K DHN E ML D FR PE V Q P L NQ F	860	Chlamydia felis
WP_348663429.1 (+)	1	TT TV KG KAP KKSEGKT VGSLL PE LM DE S G I K DNN AE LL D FKA PE V Q P L Q F	861	Chlamydia sp. BM-2023
WP_011006940.1 (+)	1	TTGS RKT TP K KKSEGKT KTT VGALL PE LM DE A G V K DHN E MLV D F AI PE V Q P L NQ F	859	Chlamydia caviae
WP_117274723.1 (+)	1	TTG KA GKTSSKTTKTAAKKKSE VGSLL PE LM NE A G I K DHN E ML D FRAI E V Q P L NQ F	856	Chlamydia poikilotherma
WP_332389941.1 (+)	1	TTG KA KTSSKTTKT AKKKSEG VGSLL PE LM NE A G I K DQN E ML E F AI PE V Q P L NQ F	856	Chlamydia sp. 04-14
WP_332380847.1 (+)	1	TG K AAGKTSSKTTKT A K EKKTT GSLL PE LM NE A G I K DHN E ML D F AI PE V Q P L NQ F	856	unclassified Chlamydia
WP_131743875.1 (+)	1	TTG KAS KMT TTAKK EGK R K GALL PQ L SE G V Q DHN E MLL D FEAI PE V Q P L NQ F	860	Chlamydia buteonis
WP_349821239.1 (+)	1	TTG KATTKSSSKTTKTTTKKKSEGKTP TL PQ LM PE L G V Q DHN E MLL D FAAI PE V Q P L NQ F S	862	Chlamydia abortus
AEG86012.1 (+)	1	TTG KATTKSSSKTTKTTAKKKKSEGKTP TL PQ LM PE G V Q DHN E MLL E FEAI PE V Q P L NQ F S	855	Chlamydia psittaci C19/98
WP_139415003.1 (+)	1	TTTG KATTKSSSKTTKTTTKKKSEGKTP TL PQ LM PE L G V Q DHN E MLL D FAAI PE V Q P L NQ F S	862	Chlamydia abortus
WP_213241861.1 (+)	1	TG K ATAKTSSKTTKT T K EKKTS VGSLL PE LM NE A G V K DRN E ML D FRAI PE V Q P L NQ F	856	Chlamydia crocodili

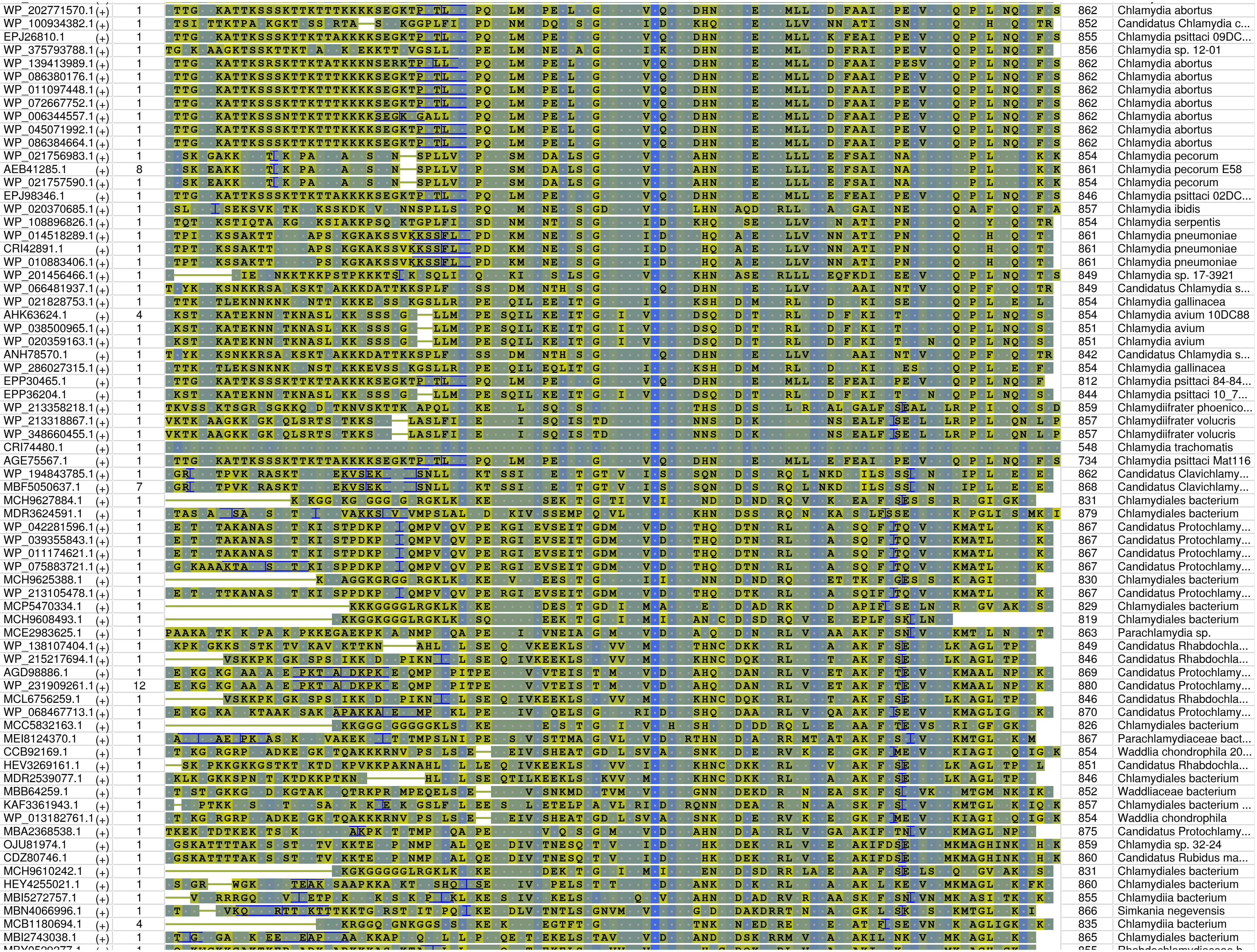

WP_202771570.1 (+) 1 862 Chlamydia abortus
WP_100934382.1 (+) 1 852 Candidatus Chlamydia c...
EPJ26810.1 (+) 1 855 Chlamydia psittaci 09DC...
WP_375793788.1 (+) 1 856 Chlamydia sp. 12-01
WP_139413989.1 (+) 1 862 Chlamydia abortus
WP_086380176.1 (+) 1 862 Chlamydia abortus
WP_011097448.1 (+) 1 862 Chlamydia abortus
WP_072667752.1 (+) 1 862 Chlamydia abortus
WP_006344557.1 (+) 1 862 Chlamydia abortus
WP_045071992.1 (+) 1 862 Chlamydia abortus
WP_086384664.1 (+) 1 862 Chlamydia abortus
WP_021756983.1 (+) 1 854 Chlamydia pecorum
AEB41285.1 (+) 8 861 Chlamydia pecorum E58
WP_021757590.1 (+) 1 854 Chlamydia pecorum
EPJ98346.1 (+) 1 846 Chlamydia psittaci 02DC...
WP_020370685.1 (+) 1 857 Chlamydia ibidis
WP_108896826.1 (+) 1 854 Chlamydia serpentis
WP_014518289.1 (+) 1 861 Chlamydia pneumoniae
CRI42891.1 (+) 1 861 Chlamydia pneumoniae
WP_010883406.1 (+) 1 861 Chlamydia pneumoniae
WP_201456466.1 (+) 1 849 Chlamydia sp. 17-3921
WP_066481937.1 (+) 1 849 Candidatus Chlamydia s...
WP_021828753.1 (+) 1 854 Chlamydia gallinacea
AHK63624.1 (+) 4 854 Chlamydia avium 10DC88
WP_038500965.1 (+) 1 851 Chlamydia avium
WP_020359163.1 (+) 1 851 Chlamydia avium
ANH78570.1 (+) 1 842 Candidatus Chlamydia s...
WP_286027315.1 (+) 1 854 Chlamydia gallinacea
EPP30465.1 (+) 1 812 Chlamydia psittaci 84-84...
EPP36204.1 (+) 1 844 Chlamydia psittaci 10_7...
WP_213358218.1 (+) 1 859 Chlamydiifrater phoenico...
WP_213318867.1 (+) 1 857 Chlamydiifrater volucris
WP_348660455.1 (+) 1 857 Chlamydiifrater volucris
CRI74480.1 (+) 1 548 Chlamydia trachomatis
AGE75567.1 (+) 1 734 Chlamydia psittaci Mat116
WP_194843785.1 (+) 1 862 Candidatus Clavichlamy...
MBF5050637.1 (+) 7 868 Candidatus Clavichlamy...
MCH9627884.1 (+) 1 831 Chlamydiales bacterium
MDR3624591.1 (+) 1 879 Chlamydiales bacterium
WP_042281596.1 (+) 1 867 Candidatus Protochlamy...
WP_039355843.1 (+) 1 867 Candidatus Protochlamy...
WP_011174621.1 (+) 1 867 Candidatus Protochlamy...
WP_075883721.1 (+) 1 867 Candidatus Protochlamy...
MCH9625388.1 (+) 1 830 Chlamydiales bacterium
WP_213105478.1 (+) 1 867 Candidatus Protochlamy...
MCP5470334.1 (+) 1 829 Chlamydiales bacterium
MCH9608493.1 (+) 1 819 Chlamydiales bacterium
MCE2983625.1 (+) 1 863 Parachlamydia sp.
WP_138107404.1 (+) 1 849 Candidatus Rhabdochla...
WP_215217694.1 (+) 1 846 Candidatus Rhabdochla...
AGD98886.1 (+) 1 869 Candidatus Protochlamy...
WP_231909261.1 (+) 12 880 Candidatus Protochlamy...
MCL6756259.1 (+) 1 846 Candidatus Rhabdochla...
WP_068467713.1 (+) 1 870 Candidatus Protochlamy...
MCC5832163.1 (+) 1 826 Chlamydiales bacterium
MEI8124370.1 (+) 1 867 Parachlamydiaceae bact...
CCB92169.1 (+) 1 854 Waddlia chondrophila 20...
HEV3269161.1 (+) 1 851 Candidatus Rhabdochla...
MDR2539077.1 (+) 1 846 Chlamydiales bacterium
MBB64259.1 (+) 1 852 Waddliaceae bacterium
KAF3361943.1 (+) 1 857 Chlamydiales bacterium ...
WP_013182761.1 (+) 1 854 Waddlia chondrophila
MBA2368538.1 (+) 1 875 Candidatus Protochlamy...
OJU81974.1 (+) 1 859 Chlamydia sp. 32-24
CDZ80746.1 (+) 1 860 Candidatus Rubidus ma...
MCH9610242.1 (+) 1 831 Chlamydiales bacterium
HEY4255021.1 (+) 1 860 Chlamydiales bacterium
MBI5272757.1 (+) 1 855 Chlamydiia bacterium
MBN4066996.1 (+) 1 866 Simkania negevensis
MCB1180694.1 (+) 4 835 Chlamydiia bacterium
MBI2743038.1 (+) 1 865 Chlamydiales bacterium

[illegible]	(+)	1	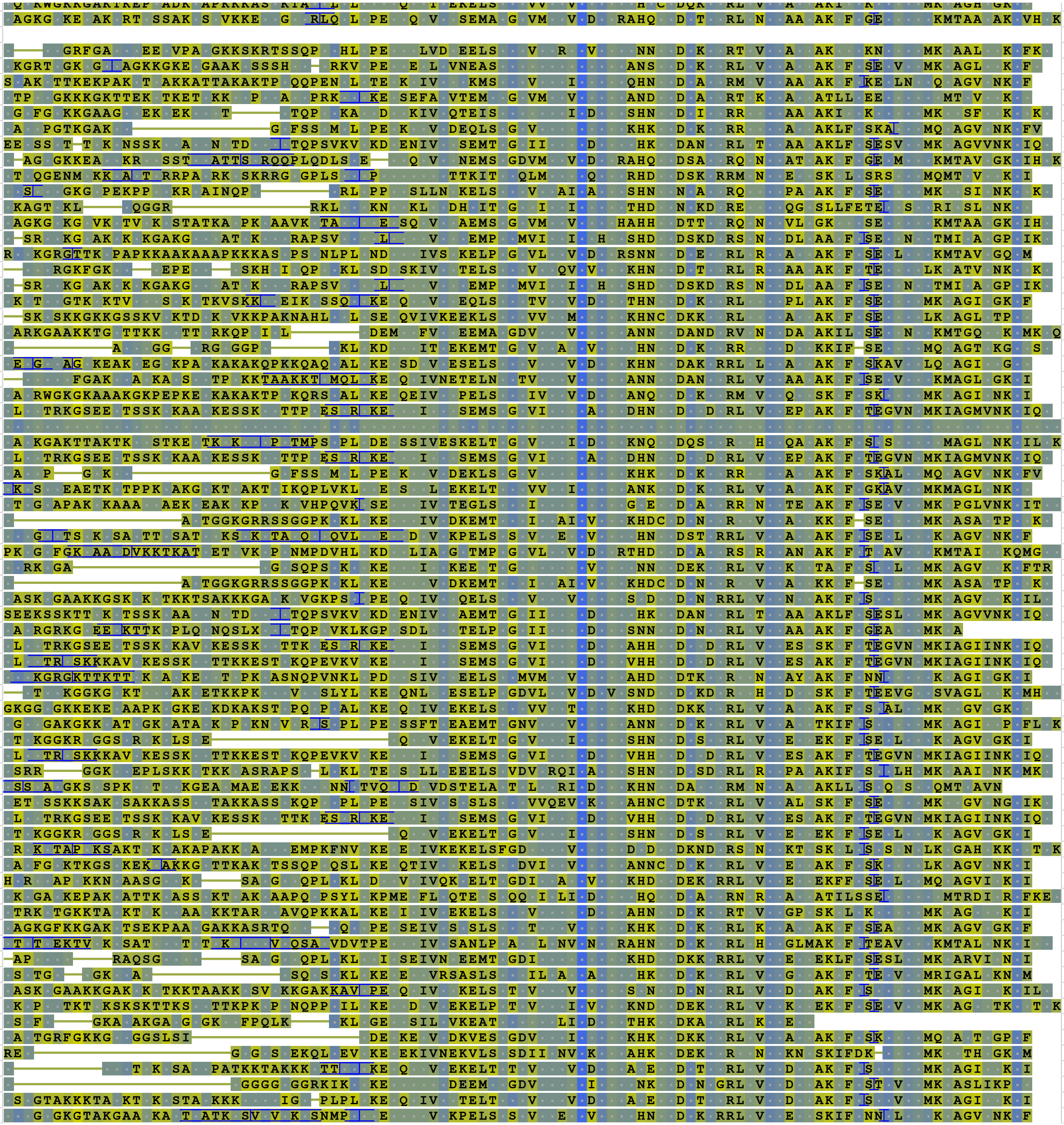	855	Rhabdochlamydiaceae b...
WP_213158404.1	(+)	1		859	Parachlamydia sp. AcF125
WP_231165312.1	(+)	1		624	Chlamydia pecorum
HEX2582512.1	(+)	1		852	Chlamydiales bacterium
MDP1608344.1	(+)	1		856	Chlamydiales bacterium
MBA3958103.1	(+)	1		867	Parachlamydiaceae bact...
MDP1835541.1	(+)	1		854	Chlamydiales bacterium
MBX7066571.1	(+)	1		849	Parachlamydiales bacteri...
HEX4839277.1	(+)	1		840	Rhabdochlamydiaceae b...
MBN9376921.1	(+)	1		866	Chlamydiales bacterium
WP_006341833.1	(+)	1		864	Parachlamydia acantha...
HSX04840.1	(+)	1		862	Rhabdochlamydiaceae b...
HSX26401.1	(+)	1		852	Chlamydiales bacterium
MEZ5315233.1	(+)	1		829	Chlamydiales bacterium
PJD97551.1	(+)	1		861	Parachlamydia sp.
MBT3393714.1	(+)	1		858	Waddliaceae bacterium
MCE5317404.1	(+)	1		853	Parachlamydia sp.
HSX10415.1	(+)	1		849	Chlamydiales bacterium
MBT3579629.1	(+)	1		858	Waddliaceae bacterium
MEI8328773.1	(+)	1		862	Chlamydiia bacterium
WP_194844625.1	(+)	1		850	Candidatus Rhabdochla...
MCB1112772.1	(+)	3		851	Chlamydiia bacterium
MCP5509411.1	(+)	3		829	Chlamydiales bacterium
HEY2810093.1	(+)	1		861	Rhabdochlamydiaceae b...
MBJ7448925.1	(+)	1		864	Parachlamydiales bacteri...
HSW87222.1	(+)	1		856	Rhabdochlamydiaceae b...
WP_166156300.1	(+)	1		861	unclassified Neochlamydia
CRH85692.1	(+)	1		458	Chlamydia trachomatis
MDP1880077.1	(+)	1		869	Parachlamydiaceae bact...
NGY95239.1	(+)	14		874	Neochlamydia sp. AcF84
HEX2579262.1	(+)	1		837	Rhabdochlamydiaceae b...
MBF8262521.1	(+)	1		856	Parachlamydiales bacteri...
MFA6916458.1	(+)	1		859	Parachlamydiales bacteri...
MCB1117265.1	(+)	3		830	Chlamydiia bacterium
MBA3237280.1	(+)	1		906	Parachlamydiaceae bact...
HEV8052804.1	(+)	6		880	Parachlamydiaceae bact...
MDX8430963.1	(+)	1		832	Candidatus Algichlamydi...
MCP5492318.1	(+)	3		830	Chlamydiia bacterium
QVL56549.1	(+)	7		861	Simkaniaceae bacterium
AGD98897.1	(+)	1		865	Neochlamydia hartmann...
QLH36937.1	(+)	3		853	Parachlamydiaceae bact...
WP_042242210.1	(+)	1		861	Neochlamydia sp. S13
KIC72453.1	(+)	14		874	Neochlamydia sp. EPS4
MBA2726565.1	(+)	1		870	Parachlamydiaceae bact...
MEX1012617.1	(+)	7		857	Waddliaceae bacterium
MBX3718257.1	(+)	1		862	Candidatus Acheromyda...
MCK4934308.1	(+)	1		857	Simkaniaceae bacterium
WP_013944621.1	(+)	7		839	Simkania negevensis
WP_044882774.1	(+)	1		861	Neochlamydia sp. EPS4
HSX37674.1	(+)	1		848	Chlamydiales bacterium
MDF2576609.1	(+)	1		865	Chlamydiales bacterium
MBX9922778.1	(+)	1		864	Rhabdochlamydiaceae b...
WP_039383557.1	(+)	1		861	Neochlamydia sp. TUME1
MCB1067455.1	(+)	7		839	Simkania sp.
WP_041018111.1	(+)	1		870	Criblamydia sequanensis
MBS3905121.1	(+)	1		856	Simkania sp.
MDN3504656.1	(+)	1		840	Candidatus Amphrikana ...
MDF2550297.1	(+)	1		855	Chlamydiales bacterium
MDN3507242.1	(+)	1		851	Candidatus Sacchlamyd...
HSX13844.1	(+)	1		851	Chlamydiales bacterium
MBA3815795.1	(+)	7		895	Parachlamydiaceae bact...
MCF7852163.1	(+)	1		841	Simkaniaceae bacterium
HAZ15965.1	(+)	1		838	Parachlamydiales bacteri...
WP_316355829.1	(+)	7		866	Candidatus Neptunochla...
MCH9631310.1	(+)	1		855	Chlamydiia bacterium
MBI3508861.1	(+)	1		822	Chlamydiia bacterium
MBP7074071.1	(+)	1		837	Rhabdochlamydiaceae b...
MFA6119465.1	(+)	1		829	Parachlamydiales bacteri...
MCB1082588.1	(+)	5		854	Chlamydiia bacterium
MBP9841130.1	(+)	1		821	Simkaniaceae bacterium
MCP5505461.1	(+)	5		854	Chlamydiales bacterium
MBA3602361.1	(+)	5		884	Parachlamydiaceae bact...

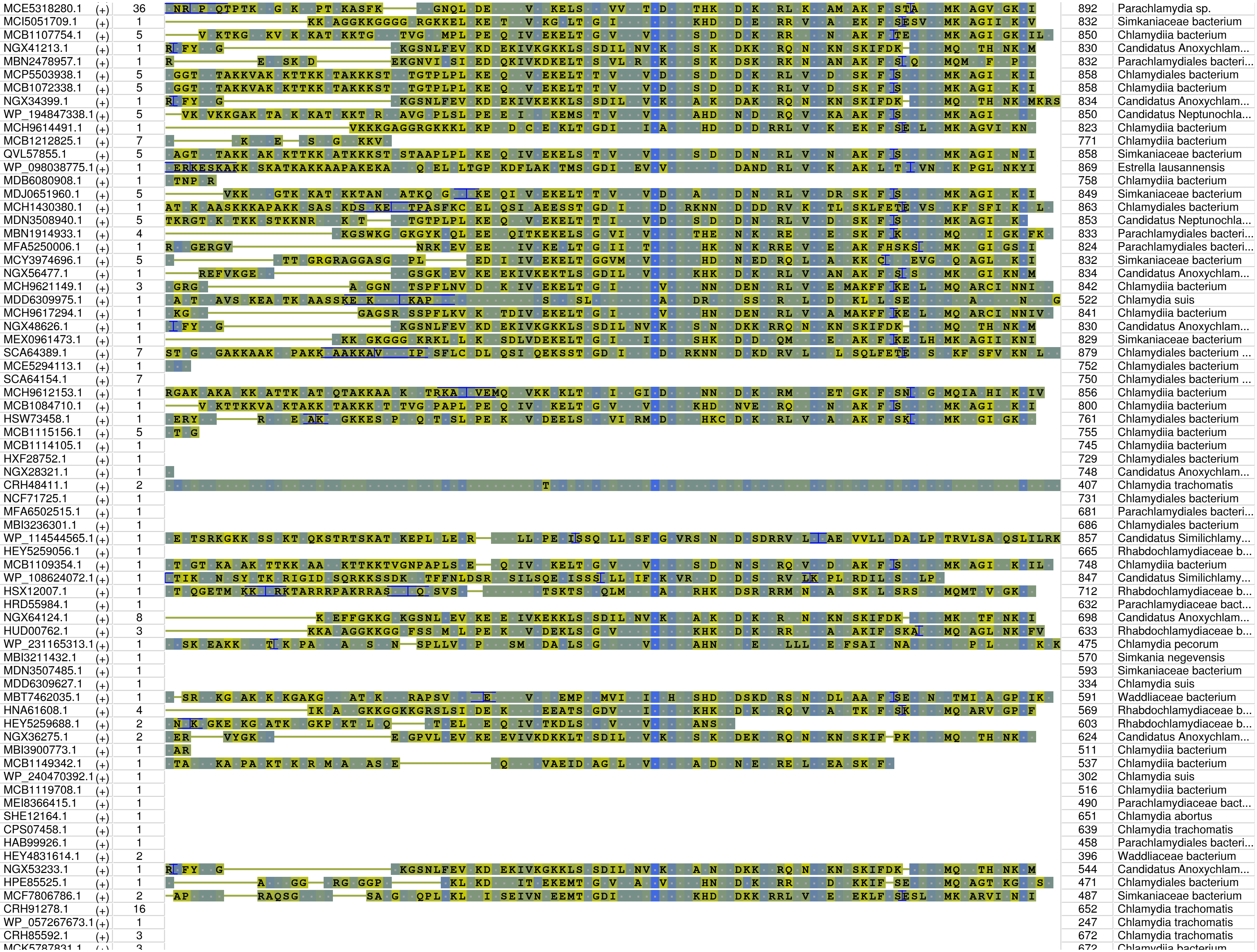
MCE5318280.1 (+) 36 892 Parachlamydia sp.
MCI5051709.1 (+) 1 832 Simkaniaceae bacterium
MCB1107754.1 (+) 5 850 Chlamydiia bacterium
NGX41213.1 (+) 1 830 Candidatus Anoxychlam...
MBN2478957.1 (+) 1 832 Parachlamydiales bacteri...
MCP5503938.1 (+) 5 858 Chlamydiales bacterium
MCB1072338.1 (+) 5 858 Chlamydiia bacterium
NGX34399.1 (+) 1 834 Candidatus Anoxychlam...
WP_194847338.1 (+) 5 850 Candidatus Neptunochla...
MCH9614491.1 (+) 1 823 Chlamydiia bacterium
MCB1212825.1 (+) 7 771 Chlamydiia bacterium
QVL57855.1 (+) 5 858 Simkaniaceae bacterium
WP_098038775.1 (+) 1 869 Estrella lausannensis
MDB6080908.1 (+) 1 758 Chlamydiia bacterium
MDJ0651960.1 (+) 5 849 Simkaniaceae bacterium
MCH1430380.1 (+) 1 863 Chlamydiales bacterium
MDN3508940.1 (+) 5 853 Candidatus Neptunochla...
MBN1914933.1 (+) 4 833 Parachlamydiales bacteri...
MFA5250006.1 (+) 1 824 Parachlamydiales bacteri...
MCY3974696.1 (+) 5 832 Simkaniaceae bacterium
NGX56477.1 (+) 1 834 Candidatus Anoxychlam...
MCH9621149.1 (+) 3 842 Chlamydiia bacterium
MDD6309975.1 (+) 1 522 Chlamydia suis
MCH9617294.1 (+) 1 841 Chlamydiia bacterium
NGX48626.1 (+) 1 830 Candidatus Anoxychlam...
MEX0961473.1 (+) 1 829 Simkaniaceae bacterium
SCA64389.1 (+) 7 879 Chlamydiales bacterium ...
MCE5294113.1 (+) 1 752 Chlamydiales bacterium
SCA64154.1 (+) 7 750 Chlamydiales bacterium ...
MCH9612153.1 (+) 1 856 Chlamydiia bacterium
MCB1084710.1 (+) 1 800 Chlamydiia bacterium
HSW73458.1 (+) 1 761 Chlamydiales bacterium
MCB1115156.1 (+) 5 755 Chlamydiia bacterium
MCB1114105.1 (+) 1 745 Chlamydiia bacterium
HXF28752.1 (+) 1 729 Chlamydiales bacterium
NGX28321.1 (+) 1 748 Candidatus Anoxychlam...
CRH48411.1 (+) 2 407 Chlamydia trachomatis
NCF71725.1 (+) 1 731 Chlamydiales bacterium
MFA6502515.1 (+) 1 681 Parachlamydiales bacteri...
MBI3236301.1 (+) 1 686 Chlamydiales bacterium
WP_114544565.1 (+) 1 857 Candidatus Similichlamy...
HEY5259056.1 (+) 1 665 Rhabdochlamydiaceae b...
MCB1109354.1 (+) 1 748 Chlamydiia bacterium
WP_108624072.1 (+) 1 847 Candidatus Similichlamy...
HSX12007.1 (+) 1 712 Rhabdochlamydiaceae b...
HRD55984.1 (+) 1 632 Parachlamydiaceae bact...
NGX64124.1 (+) 8 698 Candidatus Anoxychlam...
HUD00762.1 (+) 3 633 Rhabdochlamydiaceae b...
WP_231165313.1 (+) 1 475 Chlamydia pecorum
MBI3211432.1 (+) 1 570 Simkania negevensis
MDN3507485.1 (+) 1 593 Simkaniaceae bacterium
MDD6309627.1 (+) 1 334 Chlamydia suis
MBT7462035.1 (+) 1 591 Waddliaceae bacterium
HNA61608.1 (+) 4 569 Rhabdochlamydiaceae b...
HEY5259688.1 (+) 2 603 Rhabdochlamydiaceae b...
NGX36275.1 (+) 2 624 Candidatus Anoxychlam...
MBI3900773.1 (+) 1 511 Chlamydiia bacterium
MCB1149342.1 (+) 1 537 Chlamydiia bacterium
WP_240470392.1 (+) 1 302 Chlamydia suis
MCB1119708.1 (+) 1 516 Chlamydiia bacterium
MEI8366415.1 (+) 1 490 Parachlamydiaceae bact...
SHE12164.1 (+) 1 651 Chlamydia abortus
CPS07458.1 (+) 1 639 Chlamydia trachomatis
HAB99926.1 (+) 1 458 Parachlamydiales bacteri...
HEY4831614.1 (+) 2 396 Waddliaceae bacterium
NGX53233.1 (+) 1 544 Candidatus Anoxychlam...
HPE85525.1 (+) 1 471 Chlamydiales bacterium
MCF7806786.1 (+) 2 487 Simkaniaceae bacterium
CRH91278.1 (+) 16 652 Chlamydia trachomatis
WP_057267673.1 (+) 1 247 Chlamydia trachomatis
CRH85592.1 (+) 3 672 Chlamydia trachomatis

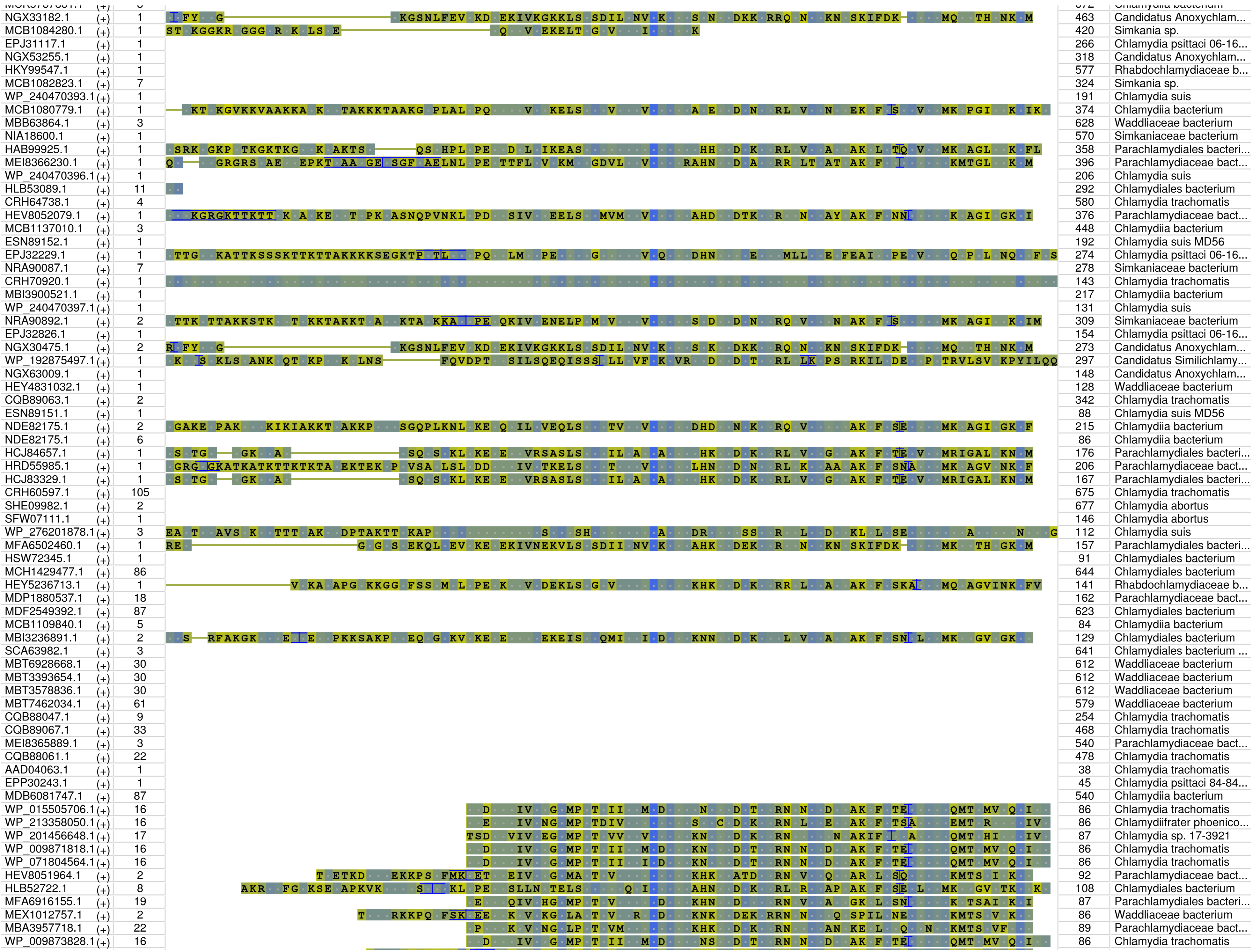
NGX33182.1 (+) 1 463 Candidatus Anoxychlam...
MCB1084280.1 (+) 1 420 Simkania sp.
EPJ31117.1 (+) 1 266 Chlamydia psittaci 06-16...
NGX53255.1 (+) 1 318 Candidatus Anoxychlam...
HKY99547.1 (+) 1 577 Rhabdochlamydiaceae b...
MCB1082823.1 (+) 7 324 Simkania sp.
WP_240470393.1 (+) 1 191 Chlamydia suis
MCB1080779.1 (+) 1 374 Chlamydiia bacterium
MBB63864.1 (+) 3 628 Waddliaceae bacterium
NIA18600.1 (+) 1 570 Simkaniaceae bacterium
HAB99925.1 (+) 1 358 Parachlamydiales bacteri...
MEI8366230.1 (+) 1 396 Parachlamydiaceae bact...
WP_240470396.1 (+) 1 206 Chlamydia suis
HLB53089.1 (+) 11 292 Chlamydiales bacterium
CRH64738.1 (+) 4 580 Chlamydia trachomatis
HEV8052079.1 (+) 1 376 Parachlamydiaceae bact...
MCB1137010.1 (+) 3 448 Chlamydiia bacterium
ESN89152.1 (+) 1 192 Chlamydia suis MD56
EPJ32229.1 (+) 1 274 Chlamydia psittaci 06-16...
NRA90087.1 (+) 7 278 Simkaniaceae bacterium
CRH70920.1 (+) 1 143 Chlamydia trachomatis
MBI3900521.1 (+) 1 217 Chlamydia bacterium
WP_240470397.1 (+) 1 131 Chlamydia suis
NRA90892.1 (+) 2 309 Simkaniaceae bacterium
EPJ32826.1 (+) 1 154 Chlamydia psittaci 06-16...
NGX30475.1 (+) 2 273 Candidatus Anoxychlam...
WP_192875497.1 (+) 1 297 Candidatus Similichlamy...
NGX63009.1 (+) 1 148 Candidatus Anoxychlam...
HEY4831032.1 (+) 1 128 Waddliaceae bacterium
CQB89063.1 (+) 2 342 Chlamydia trachomatis
ESN89151.1 (+) 1 88 Chlamydia suis MD56
NDE82175.1 (+) 2 215 Chlamydia bacterium
NDE82175.1 (+) 6 86 Chlamydia bacterium
HCJ84657.1 (+) 1 176 Parachlamydiales bacteri...
HRD55985.1 (+) 1 206 Parachlamydiaceae bact...
HCJ83329.1 (+) 1 167 Parachlamydiales bacteri...
CRH60597.1 (+) 105 675 Chlamydia trachomatis
SHE09982.1 (+) 2 677 Chlamydia abortus
SFW07111.1 (+) 1 146 Chlamydia abortus
WP_276201878.1 (+) 3 112 Chlamydia suis
MFA6502460.1 (+) 1 157 Parachlamydiales bacteri...
HSW72345.1 (+) 1 91 Chlamydiales bacterium
MCH1429477.1 (+) 86 644 Chlamydiales bacterium
HEY5236713.1 (+) 1 141 Rhabdochlamydiaceae b...
MDP1880537.1 (+) 18 162 Parachlamydiaceae bact...
MDF2549392.1 (+) 87 623 Chlamydiales bacterium
MCB1109840.1 (+) 5 84 Chlamydia bacterium
MBI3236891.1 (+) 2 129 Chlamydiales bacterium
SCA63982.1 (+) 3 641 Chlamydiales bacterium ...
MBT6928668.1 (+) 30 612 Waddliaceae bacterium
MBT3393654.1 (+) 30 612 Waddliaceae bacterium
MBT3578836.1 (+) 30 612 Waddliaceae bacterium
MBT7462034.1 (+) 61 579 Waddliaceae bacterium
CQB88047.1 (+) 9 254 Chlamydia trachomatis
CQB89067.1 (+) 33 468 Chlamydia trachomatis
MEI8365889.1 (+) 3 540 Parachlamydiaceae bact...
CQB88061.1 (+) 22 478 Chlamydia trachomatis
AAD04063.1 (+) 1 38 Chlamydia trachomatis
EPP30243.1 (+) 1 45 Chlamydia psittaci 84-84...
MDB6081747.1 (+) 87 540 Chlamydia bacterium
WP_015505706.1 (+) 16 86 Chlamydia trachomatis
WP_213358050.1 (+) 16 86 Chlamydiifrater phoenico...
WP_201456648.1 (+) 17 87 Chlamydia sp. 17-3921
WP_009871818.1 (+) 16 86 Chlamydia trachomatis
WP_071804564.1 (+) 16 86 Chlamydia trachomatis
HEV8051964.1 (+) 2 92 Parachlamydiaceae bact...
HLB52722.1 (+) 8 108 Chlamydiales bacterium
MFA6916155.1 (+) 19 87 Parachlamydiales bacteri...
MEX1012757.1 (+) 2 86 Waddliaceae bacterium
MBA3957718.1 (+) 22 89 Parachlamydiaceae bact...
WP_009873828.1 (+) 16 86 Chlamydia trachomatis

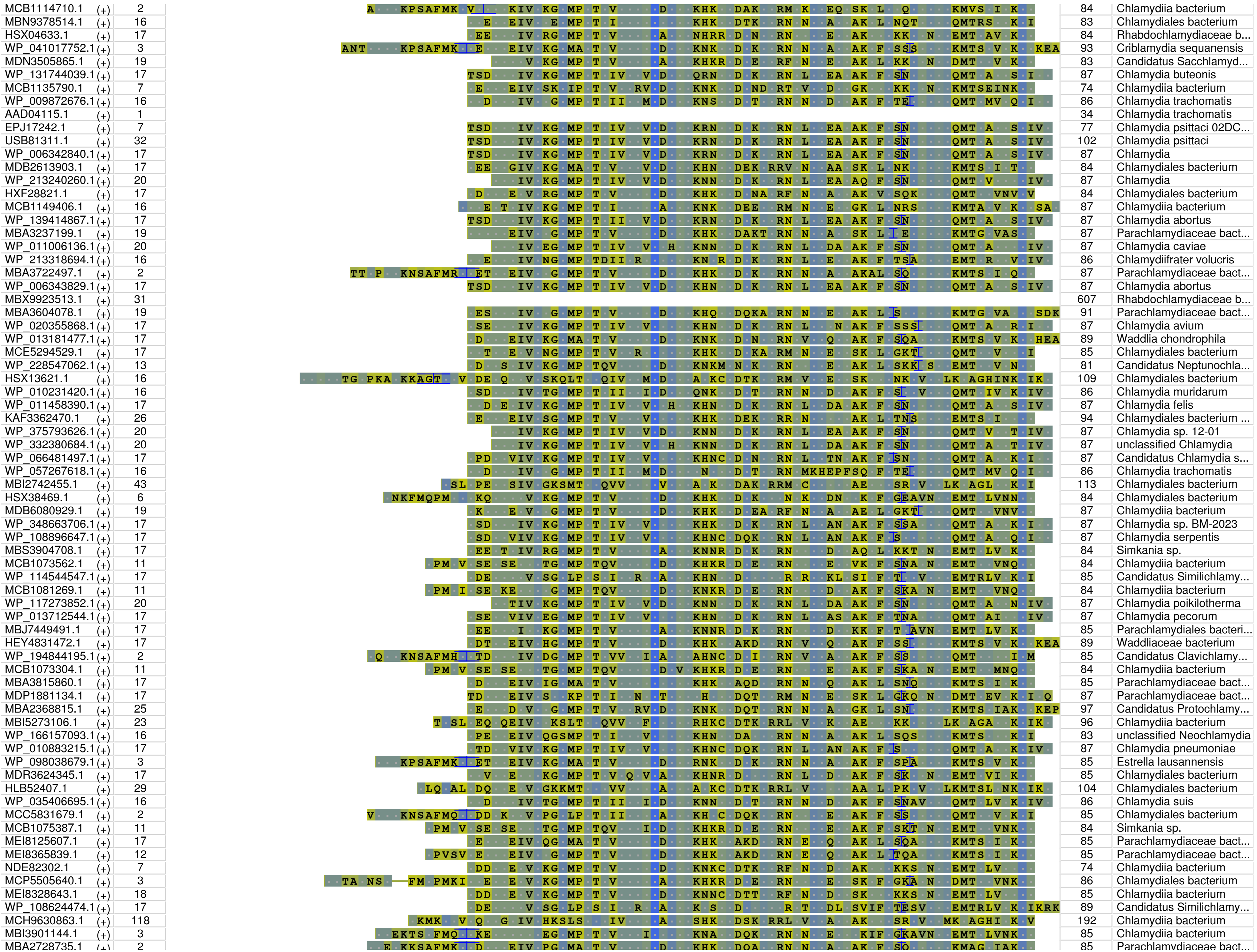
MCB1114710.1 (+) 2 84 Chlamydiia bacterium
MBN9378514.1 (+) 16 83 Chlamydiales bacterium
HSX04633.1 (+) 17 84 Rhabdochlamydiaceae b...
WP_041017752.1 (+) 3 93 Criblamydia sequanensis
MDN3505865.1 (+) 19 83 Candidatus Sacchlamyd...
WP_131744039.1 (+) 17 87 Chlamydia buteonis
MCB1135790.1 (+) 7 74 Chlamydiia bacterium
WP_009872676.1 (+) 16 86 Chlamydia trachomatis
AAD04115.1 (+) 1 34 Chlamydia trachomatis
EPJ17242.1 (+) 7 77 Chlamydia psittaci 02DC...
USB81311.1 (+) 32 102 Chlamydia psittaci
WP_006342840.1 (+) 17 87 Chlamydia
MDB2613903.1 (+) 17 84 Chlamydiales bacterium
WP_213240260.1 (+) 20 87 Chlamydia
HXF28821.1 (+) 17 84 Chlamydiales bacterium
MCB1149406.1 (+) 16 87 Chlamydiia bacterium
WP_139414867.1 (+) 17 87 Chlamydia abortus
MBA3237199.1 (+) 19 87 Parachlamydiaceae bact...
WP_011006136.1 (+) 20 87 Chlamydia caviae
WP_213318694.1 (+) 16 86 Chlamydiifrater volucris
MBA3722497.1 (+) 2 87 Parachlamydiaceae bact...
WP_006343829.1 (+) 17 87 Chlamydia abortus
MBX9923513.1 (+) 31 607 Rhabdochlamydiaceae b...
MBA3604078.1 (+) 19 91 Parachlamydiaceae bact...
WP_020355868.1 (+) 17 87 Chlamydia avium
WP_013181477.1 (+) 17 89 Waddlia chondrophila
MCE5294529.1 (+) 17 85 Chlamydiales bacterium
WP_228547062.1 (+) 13 81 Candidatus Neptunochla...
HSX13621.1 (+) 16 109 Chlamydiales bacterium
WP_010231420.1 (+) 16 86 Chlamydia muridarum
WP_011458390.1 (+) 17 87 Chlamydia felis
KAF3362470.1 (+) 26 94 Chlamydiales bacterium ...
WP_375793626.1 (+) 20 87 Chlamydia sp. 12-01
WP_332380684.1 (+) 20 87 unclassified Chlamydia
WP_066481497.1 (+) 17 87 Candidatus Chlamydia s...
WP_057267618.1 (+) 16 86 Chlamydia trachomatis
MBI2742455.1 (+) 43 113 Chlamydiales bacterium
HSX38469.1 (+) 6 84 Chlamydiales bacterium
MDB6080929.1 (+) 19 87 Chlamydiia bacterium
WP_348663706.1 (+) 17 87 Chlamydia sp. BM-2023
WP_108896647.1 (+) 17 87 Chlamydia serpentis
MBS3904708.1 (+) 17 84 Simkania sp.
MCB1073562.1 (+) 11 84 Chlamydiia bacterium
WP_114544547.1 (+) 17 85 Candidatus Similichlamy...
MCB1081269.1 (+) 11 84 Chlamydiia bacterium
WP_117273852.1 (+) 20 87 Chlamydia poikilotherma
WP_013712544.1 (+) 17 87 Chlamydia pecorum
MBJ7449491.1 (+) 17 85 Parachlamydiales bacteri...
HEY4831472.1 (+) 17 89 Waddliaceae bacterium
WP_194844195.1 (+) 2 85 Candidatus Clavichlamy...
MCB1073304.1 (+) 11 84 Chlamydiia bacterium
MBA3815860.1 (+) 17 85 Parachlamydiaceae bact...
MDP1881134.1 (+) 17 87 Parachlamydiaceae bact...
MBA2368815.1 (+) 25 97 Candidatus Protochlamy...
MBI5273106.1 (+) 23 96 Chlamydiia bacterium
WP_166157093.1 (+) 16 83 unclassified Neochlamydia
WP_010883215.1 (+) 17 87 Chlamydia pneumoniae
WP_098038679.1 (+) 3 85 Estrella lausannensis
MDR3624345.1 (+) 17 85 Chlamydiales bacterium
HLB52407.1 (+) 29 104 Chlamydiales bacterium
WP_035406695.1 (+) 16 86 Chlamydia suis
MCC5831679.1 (+) 2 85 Chlamydiales bacterium
MCB1075387.1 (+) 11 84 Simkania sp.
MEI8125607.1 (+) 17 85 Parachlamydiaceae bact...
MEI8365839.1 (+) 12 85 Parachlamydiaceae bact...
NDE82302.1 (+) 7 74 Chlamydiia bacterium
MCP5505640.1 (+) 3 86 Chlamydiales bacterium
MEI8328643.1 (+) 18 85 Chlamydiia bacterium
WP_108624474.1 (+) 17 89 Candidatus Similichlamy...
MCH9630863.1 (+) 118 192 Chlamydiia bacterium
MBI3901144.1 (+) 3 85 Chlamydiia bacterium
MBA2728735.1 (+) 2 85 Parachlamydiaceae bact...

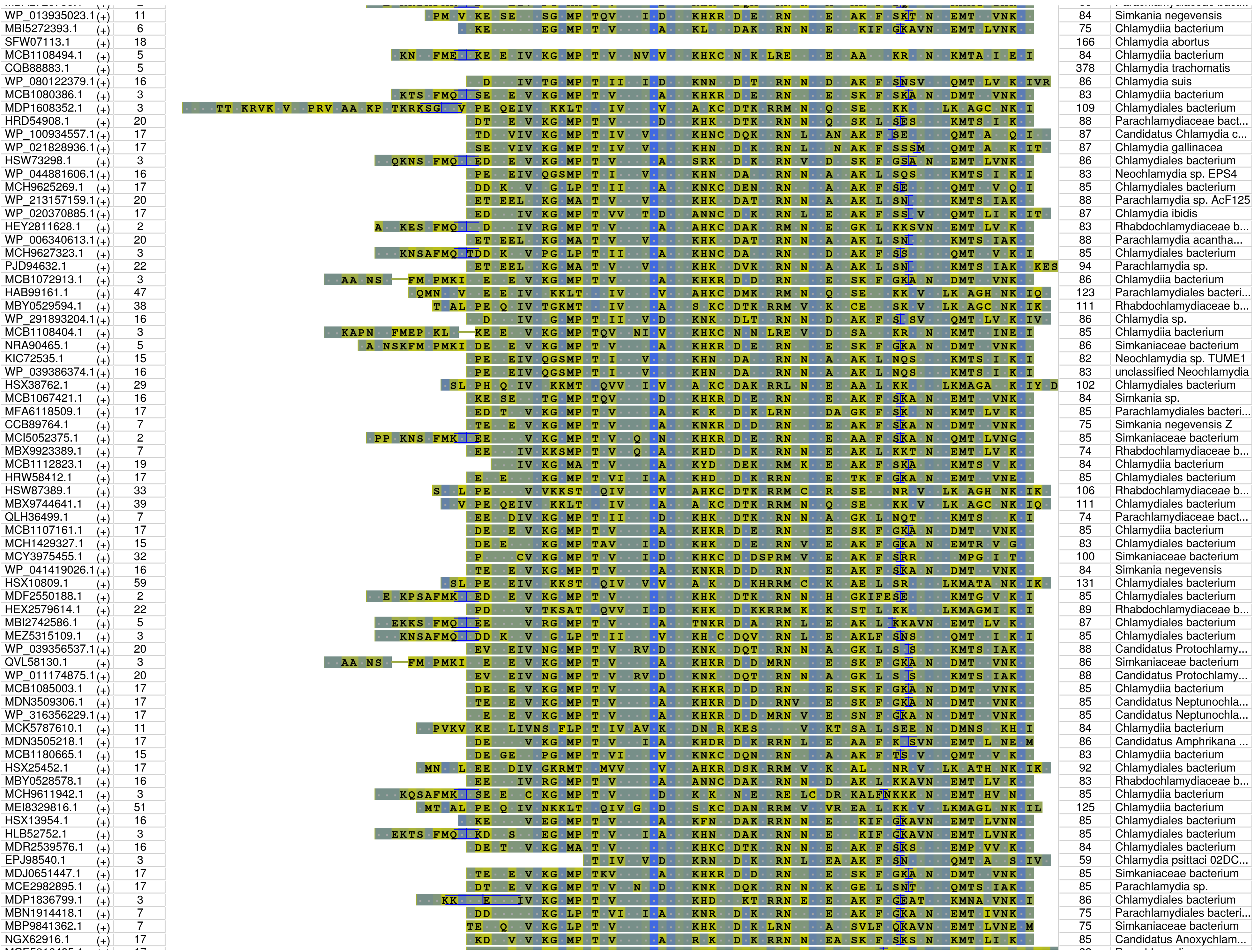

WP_013935023.1 (+) 11 84 Simkania negevensis
MBI5272393.1 (+) 6 75 Chlamydiia bacterium
SFW07113.1 (+) 18 166 Chlamydia abortus
MCB1108494.1 (+) 5 84 Chlamydia bacterium
CQB88883.1 (+) 5 378 Chlamydia trachomatis
WP_080122379.1 (+) 16 86 Chlamydia suis
MCB1080386.1 (+) 3 83 Chlamydia bacterium
MDP1608352.1 (+) 3 109 Chlamydiales bacterium
HRD54908.1 (+) 20 88 Parachlamydiaceae bact...
WP_100934557.1 (+) 17 87 Candidatus Chlamydia c...
WP_021828936.1 (+) 17 87 Chlamydia gallinacea
HSW73298.1 (+) 3 86 Chlamydiales bacterium
WP_044881606.1 (+) 16 83 Neochlamydia sp. EPS4
MCH9625269.1 (+) 17 85 Chlamydiales bacterium
WP_213157159.1 (+) 20 88 Parachlamydia sp. AcF125
WP_020370885.1 (+) 17 87 Chlamydia ibidis
HEY2811628.1 (+) 2 83 Rhabdochlamydiaceae b...
WP_006340613.1 (+) 20 88 Parachlamydia acantha...
MCH9627323.1 (+) 3 85 Chlamydiales bacterium
PJD94632.1 (+) 22 94 Parachlamydia sp.
MCB1072913.1 (+) 3 86 Chlamydia bacterium
HAB99161.1 (+) 47 123 Parachlamydiales bacteri...
MBY0529594.1 (+) 38 111 Rhabdochlamydiaceae b...
WP_291893204.1 (+) 16 86 Chlamydia sp.
MCB1108404.1 (+) 3 85 Chlamydia bacterium
NRA90465.1 (+) 5 86 Simkaniaceae bacterium
KIC72535.1 (+) 15 82 Neochlamydia sp. TUME1
WP_039386374.1 (+) 16 83 unclassified Neochlamydia
HSX38762.1 (+) 29 102 Chlamydiales bacterium
MCB1067421.1 (+) 16 84 Simkania sp.
MFA6118509.1 (+) 17 85 Parachlamydiales bacteri...
CCB89764.1 (+) 7 75 Simkania negevensis Z
MCI5052375.1 (+) 2 85 Simkaniaceae bacterium
MBX9923389.1 (+) 7 74 Rhabdochlamydiaceae b...
MCB1112823.1 (+) 19 84 Chlamydia bacterium
HRW58412.1 (+) 17 85 Chlamydiales bacterium
HSW87389.1 (+) 33 106 Rhabdochlamydiaceae b...
MBX9744641.1 (+) 39 111 Chlamydiales bacterium
QLH36499.1 (+) 7 74 Parachlamydiaceae bact...
MCB1107161.1 (+) 17 85 Chlamydia bacterium
MCH1429327.1 (+) 15 83 Chlamydiales bacterium
MCY3975455.1 (+) 32 100 Simkaniaceae bacterium
WP_041419026.1 (+) 16 84 Simkania negevensis
HSX10809.1 (+) 59 131 Chlamydiales bacterium
MDF2550188.1 (+) 2 85 Chlamydiales bacterium
HEX2579614.1 (+) 22 89 Rhabdochlamydiaceae b...
MBI2742586.1 (+) 5 87 Chlamydiales bacterium
MEZ5315109.1 (+) 3 85 Chlamydiales bacterium
WP_039356537.1 (+) 20 88 Candidatus Protochlamy...
QVL58130.1 (+) 3 86 Simkaniaceae bacterium
WP_011174875.1 (+) 20 88 Candidatus Protochlamy...
MCB1085003.1 (+) 17 85 Chlamydia bacterium
MDN3509306.1 (+) 17 85 Candidatus Neptunochla...
WP_316356229.1 (+) 17 85 Candidatus Neptunochla...
MCK5787610.1 (+) 11 84 Chlamydia bacterium
MDN3505218.1 (+) 17 86 Candidatus Amphrikana ...
MCB1180665.1 (+) 15 83 Chlamydia bacterium
HSX25452.1 (+) 17 92 Chlamydiales bacterium
MBY0528578.1 (+) 16 83 Rhabdochlamydiaceae b...
MCH9611942.1 (+) 3 85 Chlamydiia bacterium
MEI8329816.1 (+) 51 125 Chlamydiia bacterium
HSX13954.1 (+) 16 85 Chlamydiales bacterium
HLB52752.1 (+) 3 85 Chlamydiales bacterium
MDR2539576.1 (+) 16 84 Chlamydiales bacterium
EPJ98540.1 (+) 3 59 Chlamydia psittaci 02DC...
MDJ0651447.1 (+) 17 85 Simkaniaceae bacterium
MCE2982895.1 (+) 17 85 Parachlamydia sp.
MDP1836799.1 (+) 3 86 Chlamydiales bacterium
MBN1914418.1 (+) 7 75 Parachlamydiales bacteri...
MBP9841362.1 (+) 7 75 Simkaniaceae bacterium
NGX62916.1 (+) 17 85 Candidatus Anoxychlam...

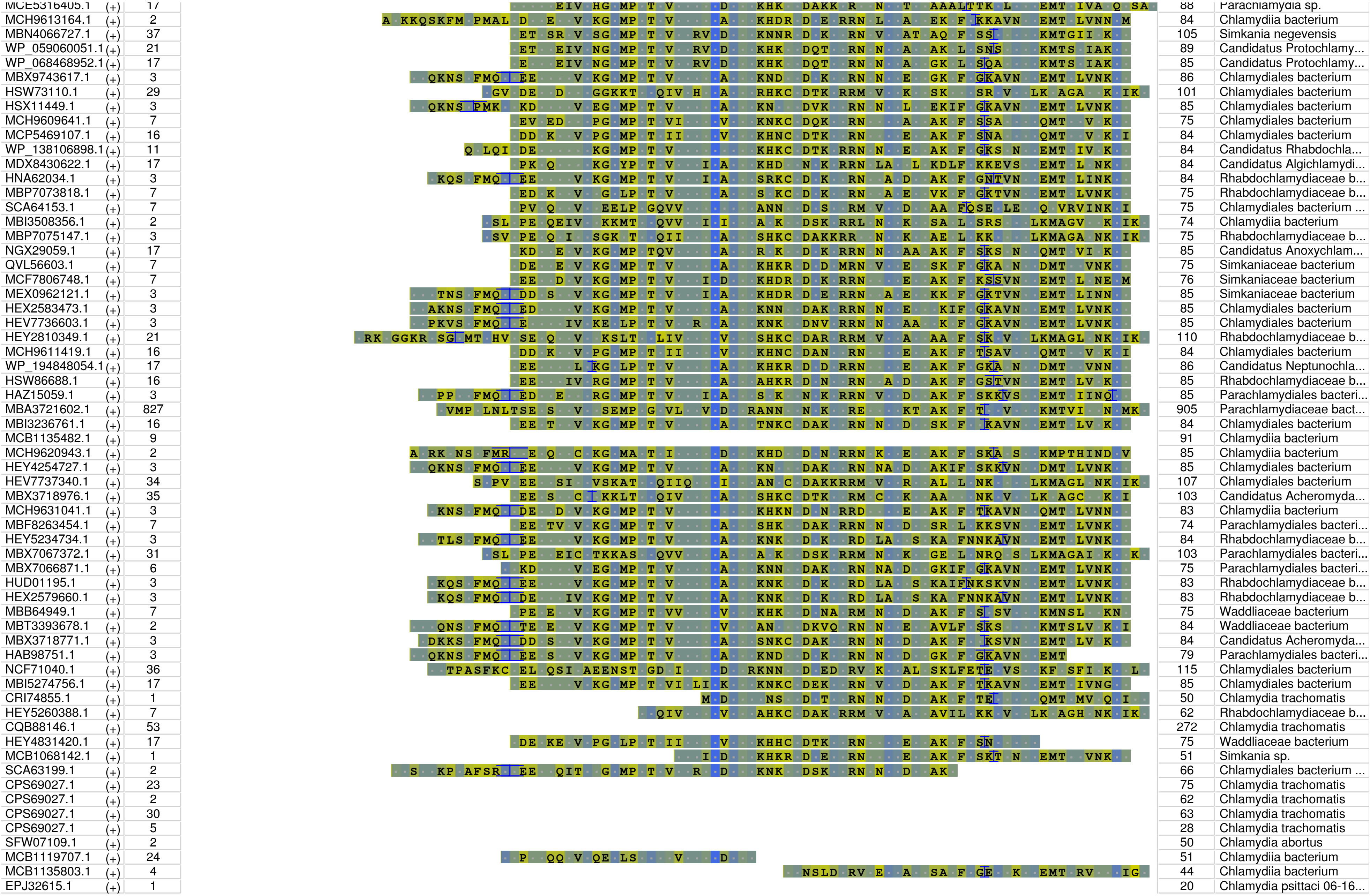
MCE5316405.1 (+) 17 88 Parachlamydia sp.
MCH9613164.1 (+) 2 84 Chlamydiia bacterium
MBN4066727.1 (+) 37 105 Simkania negevensis
WP_059060051.1 (+) 21 89 Candidatus Protochlamy...
WP_068468952.1 (+) 17 85 Candidatus Protochlamy...
MBX9743617.1 (+) 3 86 Chlamydiales bacterium
HSW73110.1 (+) 29 101 Chlamydiales bacterium
HSX11449.1 (+) 3 85 Chlamydiales bacterium
MCH9609641.1 (+) 7 75 Chlamydiales bacterium
MCP5469107.1 (+) 16 84 Chlamydiales bacterium
WP_138106898.1 (+) 11 84 Candidatus Rhabdochla...
MDX8430622.1 (+) 17 84 Candidatus Algichlamydi...
HNA62034.1 (+) 3 84 Rhabdochlamydiaceae b...
MBP7073818.1 (+) 7 75 Rhabdochlamydiaceae b...
SCA64153.1 (+) 7 75 Chlamydiales bacterium ...
MBI3508356.1 (+) 2 74 Chlamydiia bacterium
MBP7075147.1 (+) 3 75 Rhabdochlamydiaceae b...
NGX29059.1 (+) 17 85 Candidatus Anoxychlam...
QVL56603.1 (+) 7 75 Simkaniaceae bacterium
MCF7806748.1 (+) 7 76 Simkaniaceae bacterium
MEX0962121.1 (+) 3 85 Simkaniaceae bacterium
HEX2583473.1 (+) 3 85 Chlamydiales bacterium
HEV7736603.1 (+) 3 85 Chlamydiales bacterium
HEY2810349.1 (+) 21 110 Rhabdochlamydiaceae b...
MCH9611419.1 (+) 16 84 Chlamydiales bacterium
WP_194848054.1 (+) 17 86 Candidatus Neptunochla...
HSW86688.1 (+) 16 85 Rhabdochlamydiaceae b...
HAZ15059.1 (+) 3 85 Parachlamydiales bacteri...
MBA3721602.1 (+) 827 905 Parachlamydiaceae bact...
MBI3236761.1 (+) 16 84 Chlamydiales bacterium
MCB1135482.1 (+) 9 91 Chlamydiia bacterium
MCH9620943.1 (+) 2 85 Chlamydiia bacterium
HEY4254727.1 (+) 3 85 Chlamydiales bacterium
HEV7737340.1 (+) 34 107 Chlamydiales bacterium
MBX3718976.1 (+) 35 103 Candidatus Acheromyda...
MCH9631041.1 (+) 3 83 Chlamydiia bacterium
MBF8263454.1 (+) 7 74 Parachlamydiales bacteri...
HEY5234734.1 (+) 3 84 Rhabdochlamydiaceae b...
MBX7067372.1 (+) 31 103 Parachlamydiales bacteri...
MBX7066871.1 (+) 6 75 Parachlamydiales bacteri...
HUD01195.1 (+) 3 83 Rhabdochlamydiaceae b...
HEX2579660.1 (+) 3 83 Rhabdochlamydiaceae b...
MBB64949.1 (+) 7 75 Waddliaceae bacterium
MBT3393678.1 (+) 2 84 Waddliaceae bacterium
MBX3718771.1 (+) 3 84 Candidatus Acheromyda...
HAB98751.1 (+) 3 79 Parachlamydiales bacteri...
NCF71040.1 (+) 36 115 Chlamydiales bacterium
MBI5274756.1 (+) 17 85 Chlamydiales bacterium
CRI74855.1 (+) 1 50 Chlamydia trachomatis
HEY5260388.1 (+) 7 62 Rhabdochlamydiaceae b...
CQB88146.1 (+) 53 272 Chlamydia trachomatis
HEY4831420.1 (+) 17 75 Waddliaceae bacterium
MCB1068142.1 (+) 1 51 Simkania sp.
SCA63199.1 (+) 2 66 Chlamydiales bacterium ...
CPS69027.1 (+) 23 75 Chlamydia trachomatis
CPS69027.1 (+) 2 62 Chlamydia trachomatis
CPS69027.1 (+) 30 63 Chlamydia trachomatis
CPS69027.1 (+) 5 28 Chlamydia trachomatis
SFW07109.1 (+) 2 50 Chlamydia abortus
MCB1119707.1 (+) 24 51 Chlamydia bacterium
MCB1135803.1 (+) 4 44 Chlamydia bacterium
EPJ32615.1 (+) 1 20 Chlamydia psittaci 06-16...

Table S2. Strains and plasmids used in this study

Strains	Relevant Details	Reference
C. trachomatis		
L2/434/Bu	Bubo isolate from human with lymphogranuloma venereum (LGV).	ATCC VR-902B™
L2/*topA*-kd	Transformed LGV/L2 with a CRISPRi plasmid encoding *topA*-specific crRNA and P*tet*-directed synthesis of dCas12 protein	Shen et al, 2024 (1)
L2/Nt	Transformed LGV/L2 as a vector control	Shen et al, 2024
L2/*topA*-kdcom	Transformed LGV/L2 with a plasmid encoding *topA-his6* for complementation	Shen et al, 2024
L2/*topA*H6	Transformed LGV/L2 with a plasmid encoding *topA-his6*	Shen et al, 2024
L2/*topAΔC*	Transformed LGV/L2 with a plasmid encoding *topAΔC-his6*	This study
L2/e.v.	Transformed LGV/L2 with a vector control for *topAΔC-his6* expression	This study
E. coli		
DH5α	Host cell for cloning<br>F- *Φ80lacZΔM15 Δ(lacZYA-argF) U169 recA1 endA1 hsdR17(rk-, mk+) phoA supE44 thi-1 gyrA96 relA1 λ-*	New England Biolabs
VS111-K2	F^-*, λ-, ΔtopA75, zci-2234::cat, Δfnr-267, rph-1*	Yale *E. coli* Genetic Resource Center
AS17	[F^-*topA17(am)* pLL1(*TetsupD43,74*)]	Wang et al, 2002 (2)
BL21(DE3)	Host cells for protein expression<br>*fhuA2 [lon] ompT gal (λ DE3) [dcm] ∆hsdS λ DE3 = λ sBamHIo ∆EcoRI-B nt::(lacI::PlacUV5::T7 gene1) i21 ∆nin5*	New England Biolabs
C41(DE3)	F- *ompT hsdSB (rB- mB-) gal dcm* (*DE3*)	Lucigen
**Plasmid**		
pBOMBLs	vector control (Spe^r) for pBOMBLs-*topAH*	Shen et al, 2024
pBOMBLs-*topAH*	P_{tet}-directed synthesis of chlamydial *topA* (Spe^r)	Shen et al, 2024
pBOMBE*topAΔC*	P_{tet}/riboswitch-directed synthesis of chlamydial *topA* tagged with 6xHis (Spe^r)	This study
pBOMBE	vector control (Spe^r) for pBOMBE*topAΔCH*	This study
pET28-CtTopA	P_{T7}-directed synthesis of chlamydial TopA (Kan^r)	This study
pET28a(+)	vector control (Kan^r) for pET28-CtTopA and pLIC-EcTOP	Novagen
pLIC-EcTOP	P_{T7}-directed synthesis of *E. coli* TopA (Kan^r)	Tan et al, 2015 (3)
2O-T-MtbTOP1	P_{T7}-directed synthesis of *M. tuberculosis* TopA (Amp^r)	Ferdous et al, 2023(4)

References

1. **Shen L, Gao L, Swoboda AR, Ouellette SP.** 2024. Targeted repression of *topA*by CRISPRi reveals a critical function for balanced DNA topoisomerase I activity in the *Chlamydia trachomatis* developmental cycle. mBio **15:**e02584-02523.
2. **Wang Y, Lynch AS, Chen SJ, Wang JC.** 2002. On the molecular basis of the thermal sensitivity of an *Escherichia coli topA* mutant. J Biol Chem **277:**1203-1209.
3. **Tan K, Zhou Q, Cheng B, Zhang Z, Joachimiak A, Tse-Dinh YC.** 2015. Structural basis for suppression of hypernegative DNA supercoiling by *E. coli* topoisomerase I. Nucleic Acids Res **43:**11031-11046.
4. **Ferdous S, Dasgupta T, Annamalai T, Tan K, Tse-Dinh YC.** 2023. The interaction between transport-segment DNA and topoisomerase IA-crystal structure of MtbTOP1 in complex with both G- and T-segments. Nucleic Acids Res **51:**349-364.
